# Modeling the combined effect of RNA-binding proteins and microRNAs in post-transcriptional regulation

**DOI:** 10.1093/nar/gkw048

**Published:** 2016-02-02

**Authors:** Saber HafezQorani, Atefeh Lafzi, Ruben G. de Bruin, Anton Jan van Zonneveld, Eric P. van der Veer, Yeşim Aydın Son, Hilal Kazan

**Affiliations:** 1 Graduate School of Informatics, Department of Health Informatics, Middle East Technical University, Üniversiteler Mahallesi, Dumlupınar Bulvarı, No:1, 06800 Ankara, Turkey; 2 Einthoven Laboratory of Experimental Vascular Medicine, Albinusdreef 2, 2333 ZA Leiden, The Netherlands; 3 Department of Nephrology, Albinusdreef 2, 2333 ZA Leiden, The Netherlands; 4 Department of Computer Engineering, Antalya International University, Çıplaklı Mahallesi, Farabi Caddesi No:23, 07190 Döşemealtı, Antalya, Turkey

## Abstract

Recent studies show that RNA-binding proteins (RBPs) and microRNAs (miRNAs) function in coordination with each other to control post-transcriptional regulation (PTR). Despite this, the majority of research to date has focused on the regulatory effect of individual RBPs or miRNAs. Here, we mapped both RBP and miRNA binding sites on human 3′UTRs and utilized this collection to better understand PTR. We show that the transcripts that lack competition for HuR binding are destabilized more after HuR depletion. We also confirm this finding for PUM1(2) by measuring genome-wide expression changes following the knockdown of PUM1(2) in HEK293 cells. Next, to find potential cooperative interactions, we identified the pairs of factors whose sites co-localize more often than expected by random chance. Upon examining these results for PUM1(2), we found that transcripts where the sites of PUM1(2) and its interacting miRNA form a stem-loop are more stabilized upon PUM1(2) depletion. Finally, using dinucleotide frequency and counts of regulatory sites as features in a regression model, we achieved an AU-ROC of 0.86 in predicting mRNA half-life in BEAS-2B cells. Altogether, our results suggest that future studies of PTR must consider the combined effects of RBPs and miRNAs, as well as their interactions.

## INTRODUCTION

Post-transcriptional regulation (PTR) is increasingly recognized as a complex system that controls every aspect of RNA metabolism. Dysregulation of PTR mechanisms is associated with many diseases such as neurodegenerative disorders, cancer and atherosclerosis (1). PTR is mediated by the interactions of trans-factors such as RNA-binding proteins (RBPs) and microRNAs (miRNAs) with cis-acting elements located in mRNAs. Characterization of these interactions is the first step to better understanding PTR and diseases associated with defective PTR.

RBPs critically regulate (pre-)mRNA splicing, localization, degradation and translation by binding to short sequences and/or structure motifs in target (pre)-mRNAs (2). Eukaryotic genomes encode for more than 850 RBPs (3,4); however, the (pre-)mRNA targets of most RBPs remain uncharacterized. The lack of motifs for the majority of RBPs prevents the analysis of PTR mechanisms. Recently developed experimental methods provide great promise in filling this gap by expanding our knowledge of RBP targets. UV cross-linking and immunoprecipitation (CLIP) based approaches have been designed to capture the *in vivo* binding sites of RBPs (5). Photoactivatable-ribonucleoside-enhanced CLIP (PARCLIP) is a widely used modification of the CLIP protocol where photoreactive nucleosides are introduced to generate characteristic sequence changes upon crosslinking (6). These mutations make it possible to distinguish between direct and indirect interactions with (pre-)mRNA targets. CLIP experiments have been performed for only a handful of RBPs, though this number is increasing rapidly. *In vitro* methods have also been used in identifying the binding specificities of RBPs. In particular, RNAcompete is a high-throughput array-based method that has been recently used to characterize the binding specificities of 207 RBPs from 20 diverse eukaryotes (7). This compendium, together with CLIP-determined binding sites, form an invaluable resource to investigate RBP-regulated mechanisms. miRNAs, an important class of non-coding RNAs, also interact with mRNAs and enhance either transcript degradation or translational repression based on sequence complementarity, effectively downregulating gene expression. Computational methods such as TargetScan (8) and PicTar (9) are commonly used programs that enable the prediction of miRNA targets. Additionally, Ago-CLIP experiments can be used to identify the miRNA sites on mRNAs *in vivo*. However, the identity of the miRNA(s) binding to an AGO cross-linked region is unknown and needs to be inferred by further computational analysis. Recent studies have shown that mRNA fate is controlled by the complex interplay of RBPs and miRNAs (10–12). An interesting example of this phenomenon is the cooperation between the RBP PUM1 and the miRNAs miR-221/222 on the 3′UTR of the tumor suppressor gene p27, where PUM1 was found to induce a conformational switch that enhanced miR-binding and repression of p27 (13). Similarly, the nuclear RBP HNRNPL was found to undergo a hypoxia-induced translocation to the cytoplasm where it competes with several miRNAs for access to the VEGFA 3′UTR through a CA-rich element, enhancing the stability of the transcript (14). Despite several similar examples of such interactions, the number of studies that have looked at the combined effect of RBPs and miRNAs in PTR is limited. Mukharjee *et al*. analyzed the combinatorial regulation by HuR and miRNAs (15). They found that the existence of overlapping miRNA sites has no effect on HuR-mediated regulation of pre(mRNAs), but that the existence of overlapping HuR sites is capable of relieving miRNA-mediated repression. Also, Jiang *et al*. investigated the interactions between a limited set of RBPs and miRNAs by looking at the positional relationship of motif occurrences (16). To the best of our knowledge, a comprehensive study of cooperative and competitive interactions between pairs of factors (i.e. all RBPs and miRNAs) is still lacking.

In this study, we mapped the RBP and miRNA target sites on human 3′UTRs by combining computational predictions with CLIP-determined peaks. We also incorporated the conservation and secondary structure context of RBP and miRNA binding sites. Such properties of target sites are well characterized for miRNAs, but relatively little is known about the functionality of RBP sites with varying degrees of accessibility and conservation. As such, we focused on two RBPs, HuR and PUM2, and assessed how accessibility and conservation of their binding sites differ between CLIP-supported sites and other sites that are computationally predicted. Next, following HuR depletion, we showed that: (i) mRNAs with CLIP-supported HuR sites are more destabilized compared to mRNAs with computationally predicted sites; and (ii) transcripts that lack competition for HuR binding display increased destabilization, clearly illustrating the effect of competition of other factors to HuR finding. We validated these findings by specifically abrogating PUM1 and PUM2 in HEK293 cells, after which we assessed genome-wide gene expression. To our knowledge this study marks the first time that the genome-wide effects of PUM1 and PUM2 knockdown have been investigated. Next, we characterized the potential interactions between the factors (i.e. between either a pair of RBPs, a pair of miRNAs or a pair of RBP and miRNA) by finding those pairs of factors with co-occurrence of binding sites higher than expected by chance. Subsequently, we further interrogated some of the predicted interactions, and showed that the co-localization of the following pairs of factors have a functional effect: HuR and MSI1, PUM1(2) and TIA1; and miR-148b and HNRNPC. We also confirmed that transcripts where the sites of PUM1(2) and its interacting miRNA form a stem-loop are more stabilized upon PUM1(2) depletion. Finally, we used logistic regression with features compiled from the counts of sites of factors and dinucleotides to accurately predict the stability and steady-state abundance of mRNAs.

## MATERIALS AND METHODS

### Mapping RBP binding sites

To map the binding sites of RBPs, we downloaded 103 position frequency matrices (PFMs) that correspond to 85 human RBPs from the RNAcompete paper (7). These PFMs (which are of length seven or eight) are generated from the alignment of top 10 7-mers determined using all data (i.e. both setA and setB of RNAcompete pool). Rather than using these top 10 7-mers directly, we generated the top 10 n-mers from the PFMs. In this way, we were able to scan for motifs that are longer than seven. An example is the FXR1 RBP for which the PFM inferred by RNAcompete is of length eight. By using the top 10 8-mers in our motif search, we can represent the binding preferences to all eight positions of this PFM. In addition to RNAcompete motifs, we downloaded the motifs (consensus motifs and the top 10 n-mer when a PFM is available) for the following well-known RBPs from RBPDB database (17): HNRNPAB, PUM1, PUM2, ELAVL2, KHSRP, ZFP36, AUF1 and CUGBP. We downloaded human 3′UTRs from UCSC Genome Browser (on 16 February 2014) and determined the genome-wide binding sites of each RBP by finding matches to its top 10 n-mers or consensus motifs on human 3′UTR sequences. We downloaded CLIP-seq and PARCLIP data for a list of RBPs (HuR, FMR1, FUS, FXR1, FXR2, HNRNPA1, HNRNPA2B1, HNRNPC, IGF2BP1-3, LIN28, PUM2, QKI, SRSF1, TDP-43, TIA1, TIAL1) from doRINA database (18). For HuR, there exists four CLIP datasets where three of them are from HEK293 cells and one of them is from HeLa cells. We took the union of three CLIP experiments in HEK293 cells. We also downloaded the scores and rank percentiles associated with each CLIP-determined peak. In addition to these RBP specific CLIP datasets, we downloaded gPARCLIP-determined peaks (3). gPARCLIP dataset contains genome-wide protein-occupied regions bound by any RBP in HEK293 cells. However, the identity of a particular RBP that binds to a region is unknown.

**Figure 1.**
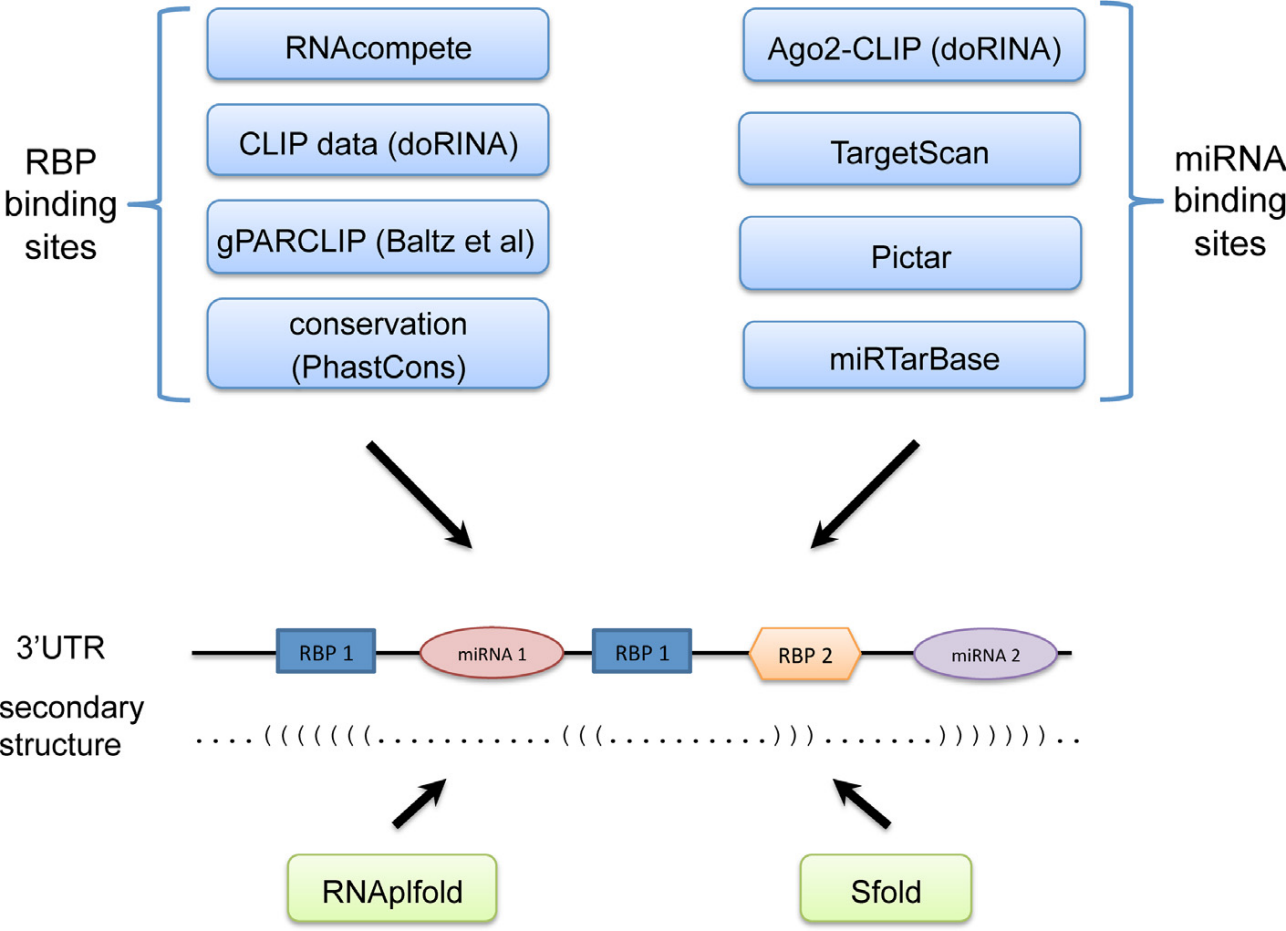
Mapping of RBP and miRNA binding sites on human 3′UTRs. RBP binding sites are mapped by leveraging RNAcompete PFMs, CLIP- and PARCLIP-determined peaks and PhastCons conservation scores. miRNA binding sites are mapped by combining TargetScan and PicTar predictions with Ago-CLIP datasets and experimentally-verified interactions in miRTarBase database. Also, the secondary structure of the 3′UTRs are predicted with RNAplfold and Sfold. In particular, accessibility of a site is calculated by RNAplfold, and the probability of a site to form a stem-loop by binding to another site is calculated by Sfold.

We first intersected the peaks identified with CLIP or gPARCLIP with human 3′UTRs. To correct for the background binding bias in CLIP-based techniques as identified by Friedersdorf *et al*. (19), we excluded parts of peaks that overlap with regions that correspond to background binding. In summary, for each putative RBP site we keep track of the following information: (i) the start and end position; (ii) a flag showing whether the site is located in a CLIP- or gPARCLIP-determined peak; and (iii) conservation score calculated as the average PhastCons score across the motif (20) (phastCons100way track downloaded from UCSC Genome Browser) (Figure 1).

### Scanning for HuR and PUM1(2) sites

We determined the HuR sites by scanning with RNAcompete top 10 n-mers (i.e. RNAcompete IDs RNCMPT00032,112,117,136,274). RNAcompete has not been performed for PUM1 or PUM2; however it has been performed for their *Drosophila* homolog Pumilio. We used the Pumilio motif as a substitute for PUM1(2) motif as they all belong to the same protein family (i.e. PUF family). The members of the PUF family contain a highly conserved RNA binding domain (RBD) known as the PUF domain (21), and the RBDs of PUM1 and PUM2 are 80 and 78% amino acid positions identical to Pumilio, respectively. Indeed, the RNAcompete motif inferred for Pumilio is very similar to the PUM1 or PUM2 motif found by *in vivo* studies such as RIP-Chip (22,23) or CLIP (6). Among the multiple RNAcompete motifs inferred for Pumilio, we chose the one (i.e. RNCMPT00104) that is most consistent with *in vivo* binding data (Supplementary Table S6 of RNAcompete paper). Additionally, we also used the consensus motifs that we downloaded from RBPDB for PUM1 and PUM2. Because PUM1 and PUM2 have similar binding specificities, we also used the PUM1 motif to scan for PUM2 sites and *vice versa*. As such, hereafter, we use PUM1(2) to represent the union of PUM1 and PUM2 binding sites.

### Mapping miRNA binding sites

To determine miRNA binding sites, we downloaded the target sites predicted by TargetScan (version 6.2) and PicTar methods (8,9) (PicTar predictions were downloaded on 20 February 2014 from doRINA database). We downloaded AGO1-4 PARCLIP, AGO2 CLIP-seq and AGO2-MNase PARCLIP datasets from doRINA database and removed background binding bias as described in the previous section. We also downloaded experimentally verified miRNA targets from miRTarBase database (24) (version 4.5). The interactions in miRTarBase database specify only whether a miRNA interacts with an mRNA without providing the exact position of the target site on the mRNA. Therefore, we assumed that a target miRNA site on an mRNA is supported by miRTarBase if that miRNA–mRNA interaction exists in the database. For each putative miRNA binding site, we keep track of the following: its start and end position, a flag showing whether the site is supported by TargetScan, PicTar or both, a flag showing whether it is supported by miRTarBase and a flag whether the site is located in CLIP- or PARCLIP-determined peaks (Figure 1).

### Compilation of datasets related to post-transcriptional regulation

The following datasets were downloaded from the supplementary material of the corresponding papers:

- **Knockdown datasets:** log fold changes (LFCs) of transcript abundance upon HuR knockdown in HEK293 and HeLa cells from (15) and (25), respectively.
- **Transfection dataset:** log fold changes (LFCs) of transcript abundance upon transfection of miR-148b in HeLa cells from (26,27).
- **Zhao dataset:** the effect of human 3′UTR segments (160-nts long) on mRNA stability and steady-state mRNA abundance in BEAS-2B human bronchial epithelial cells (28).
- **Schueler dataset:** mRNA half-lives measured in HEK293 and MCF7 cells (29). Average of the two replicate measurements is used.

The set of expressed RBPs and miRNAs can vary between different cell lines. As such, it is important to consider only those factors that are expressed in the corresponding cell line for the analysis of the datasets above. We used the following sources to define the set of expressed RBPs and miRNAs in a given cell type:

- HEK293 cells:
  – RBPs: quantitative mass spectrometry results (3).
  – miRNAs: top 100 expressed miRNAs identified with small-RNA sequencing (6).
- HeLa cells:
  – RBPs: immunohistochemistry results from Human Protein Atlas database (http://www.proteinatlas.org).
  – miRNAs: top 100 expressed miRNAs identified with small-RNA sequencing (25).
- MCF7 cells:
  – RBPs: immunohistochemistry results from Human Protein Atlas database.
  – miRNAs: top 100 expressed miRNAs identified with small-RNA sequencing (30).
- BEAS-2B cells:
  – RBPs: immunohistochemistry results from Human Protein Atlas database.
  – miRNAs: top 100 expressed miRNAs identified with small-RNA sequencing (28).

Quantitative mass spectrometry data was only available for HEK293 cells. As such, for all the other cell lines, we used immunohistochemistry results from Human Protein Atlas. We checked whether this introduces any bias by calculating the overlap between sets of expressed RBPs across the cell lines. We found that the set of expressed RBPs across different cell lines are largely shared indicating that the different sources for protein expression data does not lead to any bias (Supplementary Figure S2).

### Predicting the RNA secondary structure of binding sites

To determine whether binding sites are accessible, we predicted RNA secondary structure of human 3′UTRs with RNAplfold (31). Since the flanking regions can affect secondary structure, we folded the 3′UTRs together with the upstream 200 nt-long flanking sequence from coding region. Previous studies have shown that local folding with a window length of 200 and base pair span of 150 gives optimal results compared to using other parameters or global folding (32,33). As such, we applied local folding by running RNAplfold with the parameters −W 200 and −L 150 where ‘-W’ specifies the length of the local window and ‘-L’ restricts the maximal base pair span. We used the base pair probabilities output from RNAplfold to determine accessibility. Namely, we calculated accessibility as the average probability of being unpaired across the site.

As in the example of PUM1-miR-221 (or miR-222) interplay (13), factors can act in cooperation by binding to the two sides of the same stem-loop. In this way, one of the factors can allow the other factor to bind its site by changing the secondary structure (i.e. accessibility) of the region. To identify similar potential cooperative interactions, we aimed to find the pairs of sites that are likely to form a stem. We calculated the number of base pairs that can be formed between each pair of sites by considering reverse complementarity only. We deemed the pairs of sites that have ≥5 base pairs as potential interactors. As one site can be reverse complementary to several other sites, we need to choose the partner site that is most likely to form a stem-loop. To define a quantitative metric for this purpose, we used SFOLD. We were unable to use RNAplfold as it only outputs the probability for a base to be in paired context, whereas we also need to know where this base is paired to (and with what probability). To determine this, we used SFOLD to generate 1000 sample structures from the Boltzmann distribution of all possible structures for each running window of length 200 nt. For each pair of binding sites, we considered those windows that both sites are located in, and parsed all the sampled structures of each window to calculate the average number of base pairs formed between the two sites. For instance, if the two sites are located at 300 and 350 nt away from the start of the 3′UTR, we consider 200-nt long running windows that start from positions 150 to 300. The final estimate is the average of 150 values that are themselves averages calculated from the number of base pairs in 1000 structures. Thus, the final value reflects the probability that the two sites will form a stem.

To determine the pairs of sites that could form a stem-loop, we sorted the potential interacting sites (determined by reverse complementarity only) according to SFOLD-calculated probabilities, and filtered out those that have a probability less than 0.1. We started from the top of this list and selected pairs of sites iteratively. We ignored alternative possible interacting sites of an already selected site. For example, let's assume that a particular 3′UTR contains three sites labeled as A, B and C, and each pair of these sites can form a stem-loop with more than ≥5 bp. Let's also assume that the Sfold-calculated probabilities for these pairs are as follows: A-B: 0.3, B-C: 0.6, A-C: 0.4. With our procedure, B-C pair would score the highest and get selected, whereas A-B and A-C pairs would be ignored as B and C are already considered.

### Testing for significance

We used one-tailed Mann–Whitney U test (also called Wilcoxon rank-sum test) to compare various properties (e.g. log fold change, stability) of sets of transcripts (see ‘Results’ section), and we used Wilcoxon signed-rank test to compare the area under the Receiving Operating Characteristic curve (AUROC) values of different logistic regression models.

### Motif co-occurrence analysis

Our co-occurrence analysis is based on previous work by Jiang *et al*. (16). Though, we extended this approach with two main improvements to deal with problems that can arise due to (i) factors that have similar binding motifs and (ii) homotypic clusters of binding sites. The first problem occurs because sites of factors that have similar binding motifs (e.g. HuR and AUF1) tend to cluster together artificially. To avoid this bias, we identified the factors with similar binding motifs (i.e. PFMs and consensus motifs for RBPs and seed sequences for miRNAs) using the TomTom tool with default parameters (34). For each factor, we ignored the set of factors that are found to have similar binding motifs. The second problem is specific to factors whose sites can occur in homotypic clusters. For example, we expect to find several overlapping HuR sites in an U-rich region. Naive counting can result in very high number of co-occurrences with other factors that have sites in the same window. As such, we pre-processed the sites so that only one (most upstream) of the overlapping sites of a factor is kept. Then, for each site of a factor, we calculated the number of neighboring sites of other factors in each 50-nt-window within 200-nt on either side of the site. We calculated an empirical *P*-value by comparing this count to the distribution of counts obtained when factor identities (keeping the site positions fixed) are shuffled 1000 times. We performed the permutation test three times by employing different types of shuffling: (i) shuffling the factor identities of sites that are located on the same chromosome; (ii) classifying the sites into three groups based on AU-content (i.e. <3, ≥3 and <6, ≥6 ) and shuffling the factor identities of the sites in the same group; (iii) classifying the sites into 10 groups according to their relative position within the 3′UTRs and shuffling the factor identities of the sites in the same group (see (16) for more details). We converted *P*-values to *q*-values to correct for multiple testing (35), and defined interacting factors as those for which there is at least one window (among the 20 windows) with *q*-value <0.01 from all of the three permutation tests.

### Analysis of competition effects

We defined two sites as overlapping if they share at least one position in common. Based on this definition, we classified the mRNAs that contain a binding site for a factor of interest into two groups. The mRNAs where all the sites of the factor of interest are overlapping with sites of other factors are classified in the *competition* group. The mRNAs that have at least one non-overlapping site for the factor of interest are classified in the *no-competition* group.

### Predicting stability and expression using logistic regression

To run logistic regression with L2 regularization, we used the glmnet package (36) with alpha parameter set to 0. Within each cross-validation run, we ran the *cv.glmnet* function to determine the optimal lambda (i.e. regularization constant) value.

### Experimental procedures

#### Cell culture

Human Embryonic Kidney 293T Cells (HEK293T) were purchased from ATCC (CRL-3216), and cultured according to the guidelines provided. In short, cells were maintained at 37°C at 5% CO_2_ in Dulbecco's Modified Eagle's Medium (DMEM), high glucose (4500 mg/l), supplemented with Penicillin (100 units/ml), Streptomycin (100 μg/ml), Glutamine (2 mM) and 10% heat inactivated fetal calf serum. Trypsin-ethylenediaminetetraacetic acid solution was used to dissociate the cells to subcultivate the cells in a 1:8 ratio, every 3-4 days (all materials were purchased from Gibco, Life technologies).

#### siRNA transfection and RNA isolation

Smartpool siRNA's were purchased from Dharmacon/GE healthcare. Three different Smartpools were used: Pumilio1 pool (cat no: M-014179-01), Pumilio2 pool (cat no: M-014031-00) and a non-targeting control smartpool (cat no: D-001206-13). Transfections were performed using Lipofectamine2000 (Life technologies) according to the manufacturer's recommendations. siRNA pools for Pumilio 1 and Pumilio 2 were mixed and diluted to a final concentration of 50nM and transfected overnight in a 24 wells culture plate. Cells were incubated for 48 h, thereafter lysed in Trizol Reagent (Life technologies). RNA was isolated using the RNeasy Mini Kit (Qiagen, cat no: 74104) according to the manufacturer's instructions. An on-column DNA digestion was also performed to eliminate any genomic DNA contamination (Qiagen cat no: 79254). RNA concentration and purity was assessed using a NanoDrop1000 (NanoDrop Products). RNA integrity number (RIN) was assessed using a BioAnalyzer (Agilent Technologies), all RNA's used had a RIN value of 10.

#### Illumina human HT-12 v4 microarray

Genome-wide gene expression was assessed using the Illumina Inc. Human HT-12 v4 Bead Chip platform. Arrays were performed by AROS Applied Biotechnology A/S (Aarhus, Denmark) according to the manufacturer's instructions. Each array contains specific probes for more than 47 000 human transcripts. Bead-Chips were scanned using the iScan system (Ilumina Inc.) and the fluorescence intensities were analyzed and annotated using GenomeStudio software (Ilumina Inc.). Quantile normalized and log_2_ transformed data was used as input to the *lmFit* function of the limma package (37) to calculate log fold changes. Raw intensity files are deposited in GEO database.

#### cDNA synthesis and qRT-PCR

cDNA was made using M-MLV-Reverse Transcriptase and random primers according to the manufacturer's instructions (Promega). qRT-PCR was performed using SYBR-select Mastermix (Applied Biosystems/Life Technologies) on a Biorad CFX384 system. Gene-specific primer-sequences were derived from the online Primerbank (http://pga.mgh.harvard.edu/primerbank/, (38) ). Primer oligonucleotides were synthesized by Sigma Aldrich. The list of primers are available in Supplementary Table S1. The expression changes of PUM1, PUM2 and three known targets of PUM1 or PUM2 (i.e. COX10, CDKN1B, SDAD1) can be found in Supplementary Figure S2.

## RESULTS

### Accessibility and conservation scores of HuR and PUM2 binding sites

One approach to map the binding sites of an RBP is to scan the genome with a pre-determined motif or PWM. However, RBPs bind to only a subset of these matches *in vivo*. Therefore, it would be useful to find additional features that can help in the identification of the true binding sites. In this analysis, our goal is to assess whether accessibility or sequence conservation can distinguish the RBP binding sites that are located in CLIP-determined peaks from other potential sites that are not located in CLIP-determined peaks. For these studies, we focused on two well-characterized proteins: HuR and PUM2.

**Figure 2.**
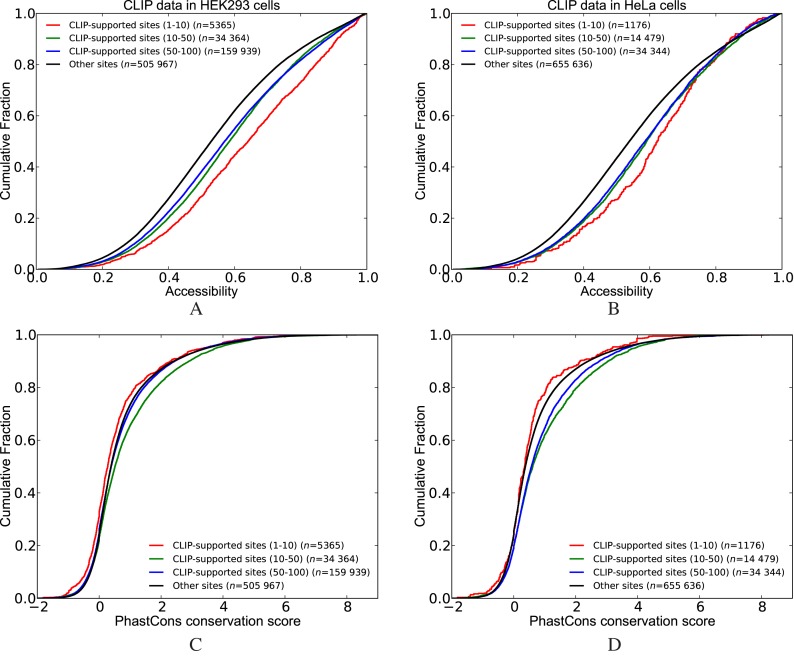
Comparison of the cumulative distribution of accessibility and conservation scores between CLIP-supported and not CLIP-supported sites (i.e. other sites) of HuR. CLIP-supported sites are classified into different groups based on the scores of the peaks that they are located in (**A** and **B**) show the accessibility scores where the CLIP dataset from HEK293 and HeLa cells was used, respectively. Similarly, (**C** and **D**) show Phastcons conservation scores. (See Supplementary Table S2 for *P*-values).

**Figure 3.**
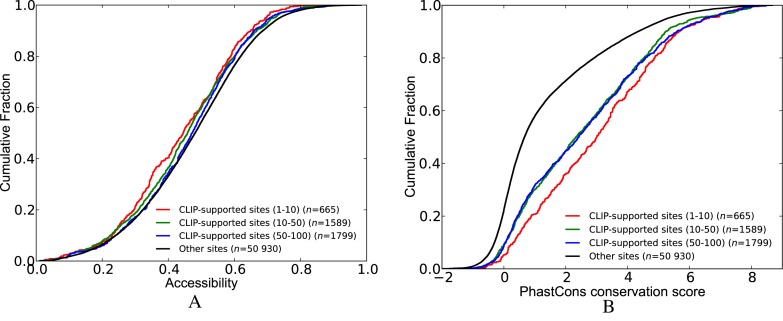
Comparison of the cumulative distribution of accessibility and conservation scores between CLIP-supported and not CLIP-supported sites (i.e. other sites) of PUM1(2). CLIP-supported sites are classified into different groups based on the scores of the peaks that they are located in. (**A**) Accessibility. (**B**) PhastCons conservation scores. (See Supplementary Table S3 for *P*-values).

First, we classified the transcripts into two groups based on whether they reside in CLIP-determined peaks (i.e. CLIP-supported sites) or not (i.e. other sites). Next, we compared the cumulative distribution of accessibility and conservation scores between these two groups (see Figures 2 and 3). In accordance with previous literature (39), we observed that the CLIP-supported HuR sites are more accessible when CLIP data from either HEK293 cells (6) or HeLa cells (25) is taken into consideration. Furthermore, grouping CLIP-supported sites according to their peak score (i.e. percentiles downloaded from doRINA database) revealed that sites that reside in peaks with higher scores are more accessible as compared to other CLIP-supported sites (Figure 2A and B). We repeated these analyses to compare the PhastCons conservation scores of binding sites (Figure 2C and D). Interestingly, for both HEK293 and HeLa cells, the CLIP-supported sites with high scores (in the highest 10% percentile) are less conserved than the sites that are not CLIP supported. On the other hand, CLIP-supported sites within 10^th^ to 50^th^ percentiles are more conserved than other sites (i.e. not CLIP supported) in both HEK293 and HeLa cells.

We observed that CLIP-supported sites of PUM1(2) are less accessible as compared to other sites. In fact, when we separate the CLIP-supported sites according to their peak scores, we found that sites located in high scoring peaks (percentile 1–10) are less accessible compared to sites located in low scoring peaks (percentile 50–100) (Figure 3A). This result is surprising as PUM1 is known to bind to single-stranded motifs (40), whereas there is no previous literature regarding a structural preference for PUM2 with mRNAs. However since PUM1 and PUM2 possess nearly identical RBDs, these studies suggested that PUM2 will also prefer accessible sites. Subsequent comparison with conservation scores enabled us to identify that the sites located in high scoring peaks are more conserved than other CLIP-supported sites or sites that are not CLIP-supported (Figure 3B).

### Analysis of RBP knockdown datasets

To gain more insight into the functional effect of the distinct properties of individual binding sites (e.g. supported by CLIP, overlapping with sites of other factors), we compiled knockdown datasets of HuR and PUM1(2). We downloaded log fold expression changes (LFCs) of transcripts upon HuR depletion in HEK293 and HeLa cells from (15) and (25), respectively. Hereafter, we name these knockdown datasets as Mukharjee and Lebedeva datasets, respectively. Mukharjee *et al*. have shown that transcripts possessing solely intronic HuR sites still show reduced expression upon HuR depletion in HEK293 cells (15). Since the aim of our study is to investigate the function of HuR sites in 3′UTRs, we filtered out those transcripts containing intronic HuR sites throughout the following analyses. This approach limits the hidden effect of intronic sites of HuR in our analysis. In the absence of existing genome-wide changes of expression upon PUM1(2) depletion, we reduced PUM1 and PUM2 expression in HEK293 cells using siRNAs, and measured the changes in genome-wide gene expression.

We analyzed each of these knockdown datasets to determine whether a correlation exists between LFCs and 3′UTR length, and between LFCs and gene expression levels (i.e. expression in mock transfected cells). For Mukharjee dataset, the Spearman correlation of LFCs versus 3′UTR length and LFCs versus expression levels were −0.06 and −0.11, respectively. Interestingly, we observed a much larger correlation when comparing LFCs with 3′UTR length (*R* = −0.22) and LFCs with expression levels (*R* = 0.14) in the Lebedeva dataset. The correlation coefficients in PUM1(2) knockdown dataset were 0.09 and −0.03 for comparisons with 3′UTR length and expression levels, respectively. Lastly, we also found that the Spearman's rank correlation coefficient of the LFCs between the two HuR knockdown datasets is only 0.1.

#### mRNAs with CLIP-supported sites show greater expression effects upon RBP knockdown

**Figure 4.**
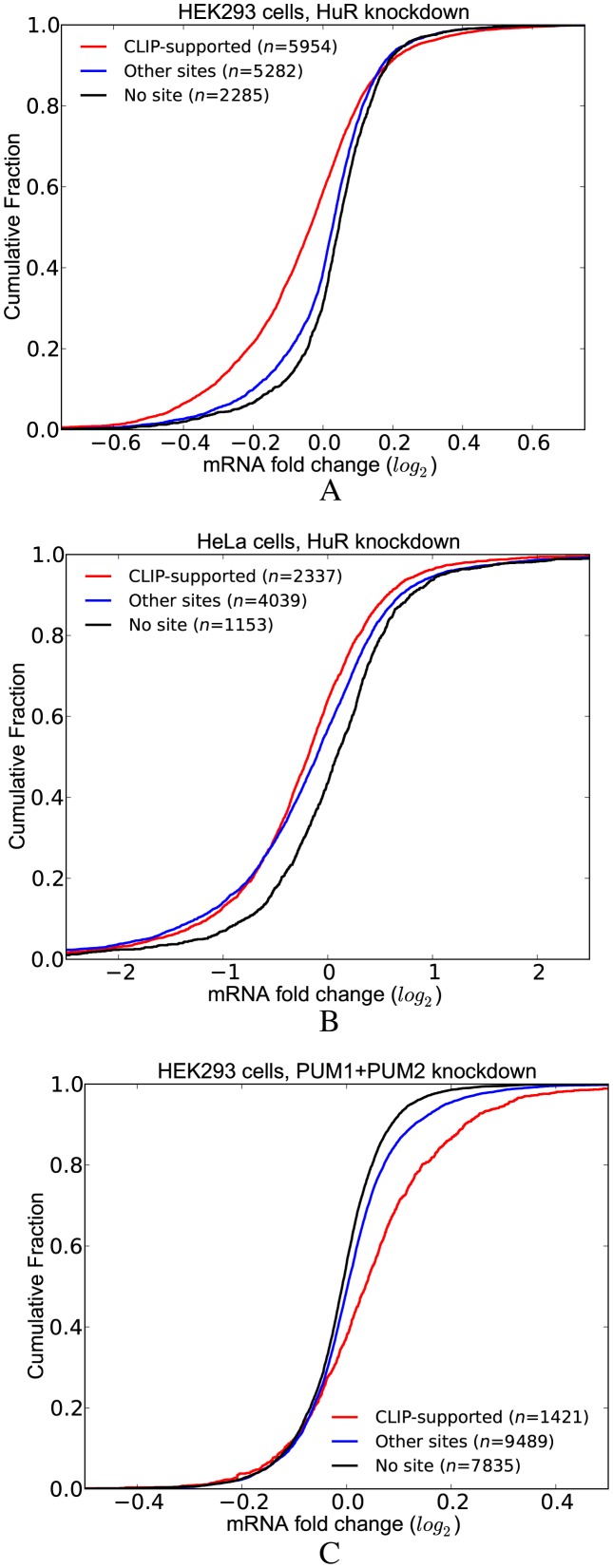
Comparison of the effect of RBP depletion on transcripts that contain CLIP-supported RBP sites against those that do not contain CLIP-supported RBP sites. X-axis shows the LFCs of transcripts upon the depletion of the RBP of interest. (**A**) HuR knockdown data in HEK293 cells. (**B**) HuR knockdown data in HeLa cells. (**C**) Double knockdown of PUM1 and PUM2 in HEK293 cells. (See Supplementary Table S4 for *P*-values).

To examine the effect of CLIP-supported sites, we classified the transcripts into three groups: (i) those that have at least one CLIP-supported HuR site (*CLIP*); (ii) those that have one or more predicted HuR sites but none of them are CLIP-supported (*other*); and (iii) those lacking HuR sites (*no site*). In Figure 4, we plotted the cumulative distribution of transcript LFCs following stratification according to these groups. If the *no site* group was deemed as baseline, we observed for both knockdown datasets that transcripts in the *CLIP* group were significantly more destabilized upon HuR depletion than transcripts in the *other* group (Figure 4A and B). As HuR is known to stabilize the expression levels of target mRNAs, these results show that CLIP-supported HuR sites more accurately predict changes in gene expression, and display greater effects than other (non-CLIP-supported) sites.

Next, we assessed whether our categorization of mRNAs in the above analysis results in groups with distinct distributions of 3′UTR length or expression levels. Indeed, for both knockdown datasets, we observed that the transcripts in the *CLIP* group have longer 3′UTRs as compared to the other groups. Subsequent comparisons of these groups in terms of expression levels yielded smaller differences compared to the differences we observed for 3′UTR length. The Mukharjee dataset revealed that the *CLIP* group have the highest expression levels while the Lebedeva dataset revealed that the *CLIP* group have lower expression levels compared to the *no site* group. To show that our findings are still evident upon removing 3′UTR length and expression levels effects, we used linear regression to predict LFCs using these two factors as features. Then, we replotted the CDF plots in Figure 4 using the residuals of this model (Supplementary Figure S3). Importantly, we observed that the transcripts in the *CLIP* group still display significantly more effect compared to the other groups in the Mukharjee dataset (Supplementary Table S11A). This difference in destabilization between the *CLIP* group and the other groups became significantly smaller for the Lebedeva dataset (Supplementary Table S11B).We indeed anticipated a larger change between the analysis with LFCs and residuals for the Lebedeva dataset due to larger positive and negative correlations between LFCs versus 3′UTR length and expression levels. In fact, when we compare our results between the two knockdown datasets before the removal of 3′ UTR length and expression levels effects, we see that the difference between the groups *CLIP* and *other*, and the difference between the groups *CLIP* and *no site* are more pronounced in the Mukharjee dataset. One explanation for this observation could be the small concordance between the two knockdown datasets. Another reason could be the existence of only a single HuR CLIP dataset in HeLa cells compared to three datasets in HEK293 cells. This might have resulted in a more comprehensive set of sites in HEK293 cells. Indeed, we found that 3865 transcripts that have CLIP-supported sites based on HEK293-CLIP data do not have any CLIP-supported site according to HeLa-CLIP data. When we plotted Figure 4B using HEK293 CLIP data rather than HeLa CLIP data we observed a much larger difference between the *CLIP* group and the other groups, and this difference is still present when we plot the same figure with residuals (Supplementary Figure S4A and B). In order to have a more robust definition of HuR-regulated transcripts we opted to utilize the HEK293 CLIP data for both knockdown datasets for subsequent analyses.

We repeated the above analysis with PUM1(2) knockdown data (Figure 4C). PUM1(2) is known to decrease the stability of its mRNA targets. As such, the depletion of PUM1(2) should result in increased expression of target mRNAs. Similar to our observations with HuR, transcripts with CLIP-supported sites displayed greater effects compared to other transcripts. This observation still holds true when we re-plotted the CDFs using the residuals that we get from the regression model fit to LFCs of the PUM1(2) dataset. (Supplementary Figure S3C, Supplementary Table S11C).

#### mRNAs with competitive sites show less effect upon RBP knockdown

So far, our analyses have focused on binding sites occupied by a single RBP. However, transcripts are known to be occupied by several RBPs and miRNAs concurrently. Here, we assessed the effect of competition of HuR and PUM1(2) with other factors. We assumed that competition exists for those RBP sites (HuR or PUM1(2)) that overlap with sites of other factors at one or more positions.

**Figure 5.**
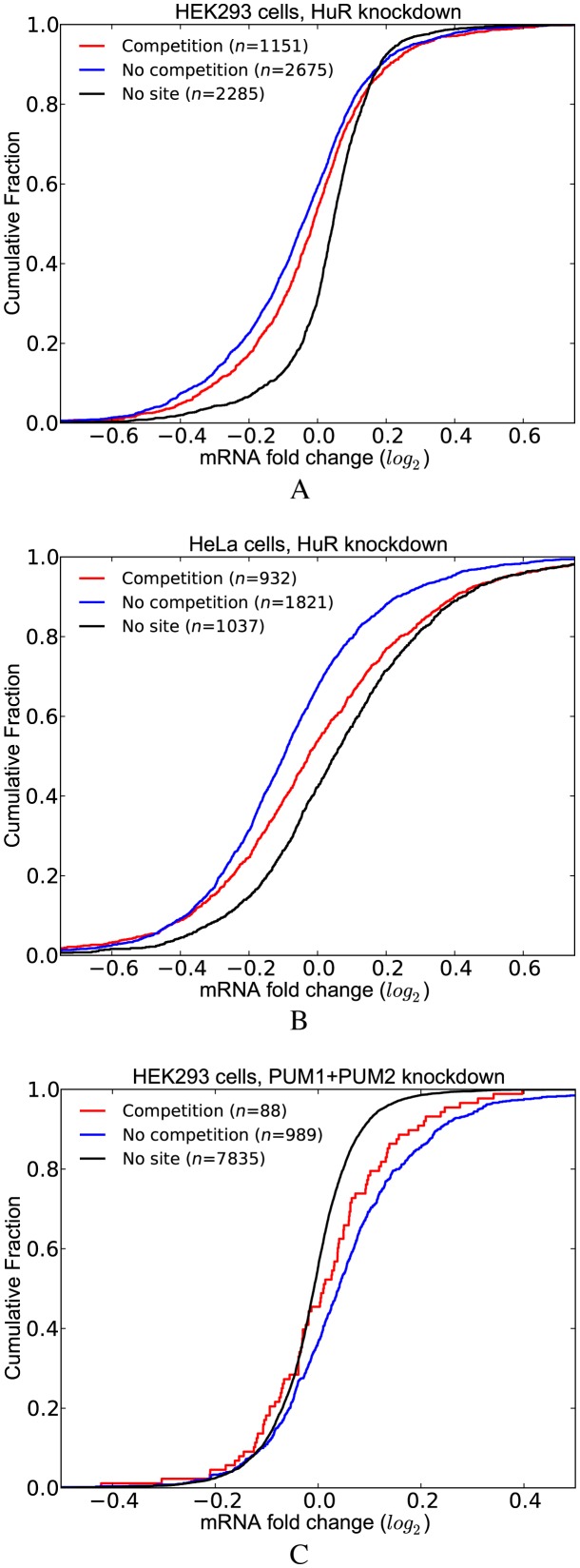
Functional outcome of the competitive effects of other factors on RBP binding and function. (**A**) HuR knockdown data in HEK293 cells. (**B**) HuR knockdown data in HeLa cells. (**C**) Double knockdown of PUM1 and PUM2 in HEK293 cells. (See Supplementary Tables S5 for *P*-values).

To examine the effect of competition in the HuR knockdown dataset, we classified the transcripts into three groups: (i) transcripts that have at least one CLIP-supported HuR site that is not overlapping with any other site (*no competition*); (ii) transcripts that have at least one CLIP-supported HuR site but the HuR sites including the CLIP-supported ones are overlapping with sites of other factors (*competition*); and (iii) transcripts that have no HuR site. Similar to the previous analysis, we initially filtered out those transcripts that contain intronic HuR sites. Figure 5 shows that the transcripts lacking competition for binding (*no competition*) display increased destabilization after HuR depletion, as compared to the transcripts in the *competition* group. Importantly, this observation holds true for HuR knockdown data from both HEK293 and HeLa cell lines (Figure 5A and B).

Subsequently, we repeated this analysis with the PUM1(2) knockdown dataset. Figure 5C compares the transcripts in the *competition* and *no competition* groups with respect to the transcripts lacking PUM1(2) binding site. These analyses revealed that transcripts in the *no competition* group are more stabilized upon depletion of PUM1(2), suggesting that competition with other factors decreases the likelihood that PUM1(2) will bind to target sites. Overall, these studies addressing the consequences of a specific reduction in HuR or PUM1(2) expression consistently indicate that the presence of overlapping binding sites for other competing factors results in distinct functional outcomes.

When we replotted the CDFs in Figure 5 with residuals, we observed the same relations between the *competition* and *no competition* groups. (Supplementary Figure S5, Supplementary Table S13). The *P*-value of the Mann–Whitney U test comparing the *competition* and *no competition* groups is slightly greater than the significance threshold (i.e. 0.06) for PUM1(2) knockdown dataset. This is likely due to the small sample size of the *competition* group. We further analyzed the effect of 3′UTR length and expression levels in this dataset by sampling transcripts with replacement from the *no competition* group to match the 3′UTR length and expression levels of the transcripts in the *competition* group. When we repeated the sampling 1000 times and calculated the *P*-values using Mann–Whitney U test, a significant difference was observed in 989 cases showing that the two groups show distinct LFC distribution (i.e. empirical *P*-value 0.011).

### Analysis of co-localization of binding sites

We set out to determine whether sites of a pair of factors co-localize within a certain distance more often than can be expected by chance. Jiang *et al*. performed a similar analysis between five RBPs and all miRNAs. Using an improved approach (See ‘Materials and Methods’ section), we extended this analysis to look at the interactions between a set of 7 RBPs and 16 miRNAs (Supplementary Table S18) with all the factors (i.e. all RBPs and miRNAs). We selected this set of RBPs and miRNAs as they have previously been shown to interact with other factors, and/or the consequences of modulating their expression on genome-wide transcript abundance has been tested. To determine the factors whose sites co-localize with the sites of a factor of interest, we counted the number of neighboring sites in four 50 nt windows upstream or downstream of each site of a factor of interest. We compared this number to the average number of co-occurrences calculated from shuffled data (details of the shuffle procedure are described in the ‘Materials and Methods’ section).

**Figure 6.**
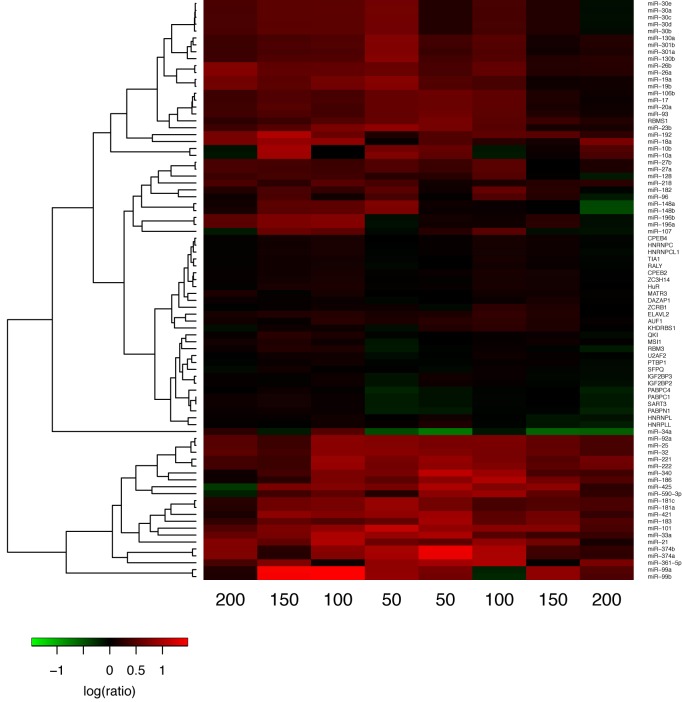
Co-occurrence of PUM1(2) sites with sites of other factors. Each row is a potential interacting factor and each column is a window. The heat map shows log-transformed enrichment ratios that are calculated as (the number of neighboring binding sites in a window/expected number of sites determined with shuffling). Minimum ratio across the three shuffle types is displayed. Row clustering is performed with hierarchical agglomerative clustering.

We classified the factors as interacting if there is at least one window (among the eight windows) with a *q*-value <0.01 from all of the three permutation tests. Based on this definition, we found that RBPs and miRNAs typically interact with many other factors. As an example, Figure 6 shows the enrichment ratios for factors that interact with PUM1(2) in each window (Enrichment ratios of other factors are available in Supplementary Figures S10-S31). As expected, factors with similar binding motifs (e.g. members of the same miRNA family or RBPs that bind to similar motifs) have similar profiles and are clustered together. We discovered that PUM1(2) sites co-localize with sites of several miRNAs. Among these are miR-221 and 222 which have been previously found to cooperate with PUM1(2) (13). Additionally, miR-374a(b) and miR-99a(b) sites are significantly over-represented near PUM1(2) sites. Overall, miRNAs that were found to interact with PUM1(2) displayed higher enrichment ratios than RBPs. Also, enrichment ratios of miRNAs vary significantly across the windows; whereas they remain consistent for RBPs. Further investigation of other factors such as HuR and PTBP1 reveals several interacting factors the majority of which are miRNAs. In contrast, the RBPs HNRNPL, QKI, IGF2BP2 and IGF2BP3 were characterized by a relatively small number of interactions. miRNAs also interact with a large number of other factors, and the scale of over- or under-representation is larger compared to RBPs (enrichment ratios and q-values are available in Supplementary Table S19-20).

Next, we utilized the knockdown datasets to investigate some of these interactions in further detail for PUM1(2) and HuR.

#### PUM1(2) have cooperative interactions with other RBPs and miRNAs

**Figure 7.**
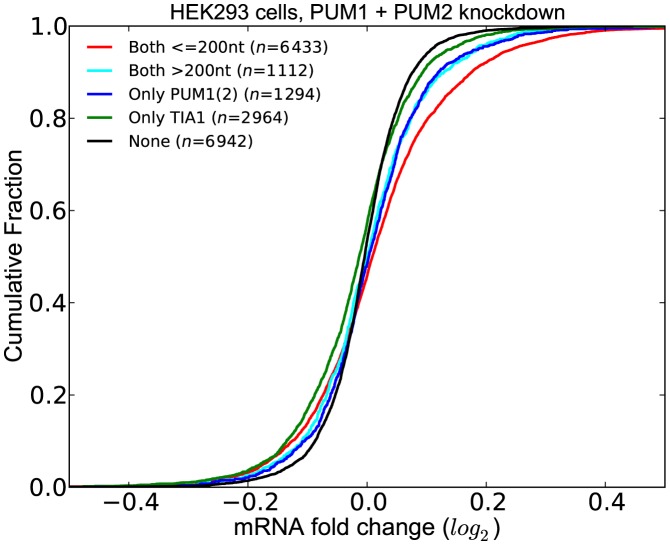
Co-occurrence of PUM1(2) and TIA1 sites have a functional effect. Transcripts that have at least one pair of PUM1(2) and TIA sites within 200 nt have significantly higher LFCs upon PUM1(2) depletion. (See Supplementary Tables S6 for *P*-values).

Among the factors that are predicted to interact with PUM1(2), we chose to investigate the interaction with TIA1, as TIA1 is a well-established repressor of target mRNAs. Given that both PUM1(2) and TIA1 serve as repressors, this enabled us to test whether transcripts containing sites for both factors have significantly lower expression compared to transcripts that have site(s) for only one of the these factors. We also considered the effect of the distance between the PUM1(2) and TIA1 sites by distinguishing the transcripts where the two sites are located within 200 nt. As shown in Figure 7, following PUM1(2) knockdown, we assessed the differential expression profile of transcripts grouped according to the presence of PUM1(2) and TIA1 sites, taking into account those PUM1(2) and TIA1 sites that are supported by CLIP or gPARCLIP datasets. Indeed, the set of transcripts where PUM1(2) and TIA1 sites are located within 200 nt show the greatest increase in expression upon PUM1(2) depletion. When the distance between the PUM1(2) and TIA1 site is >200 nt, this effect disappears. In fact, there is no statistically significant difference between the LFCs of these transcripts and the transcripts that have only PUM1(2) sites. These results indicate that the potential interaction between PUM1(2) and TIA1 occurs when their sites are proximal to one another. We observed the same results when we repeated the analysis with residuals (Supplementary Figure S6).

PUM2 has previously been found to be interacting with miRNAs (13,16). Here, we assessed whether these interactions can be recovered with our compendium of binding sites in our experimentally generated dataset where PUM1(2) expression levels were decreased. We defined the interacting miRNAs as those that have a significant *q*-value within 200 nt (upstream or downstream) of the PUM1(2) site. We then assessed the effect of co-occurrence of sites of these miRNAs with PUM1(2) sites when PUM1 and PUM2 are depleted. We classified the transcripts into five groups:

- those that do not have any PUM1(2) or miRNA sites (*None*)
- those that contain at least one CLIP-supported PUM1(2) site but do not contain any site of an interacting miRNA (*Only PUM1(2)*)
- those that contain at least one interacting miRNA site but do not contain any PUM1(2) sites (*Only miRNA*)
- those that contain at least one CLIP-supported PUM1(2) site and at least one site of an interacting miRNA (*Both (not stem-loop)*)
- those that contain at least one CLIP-supported PUM1(2) site and at least one site of an interacting miRNA with the additional constraint that the PUM1(2) site forms a stem-loop with the miRNA site (*Both (stem-loop)*) (See ‘Materials and Methods’ for details on how we predict stem-loops).

**Figure 8.**
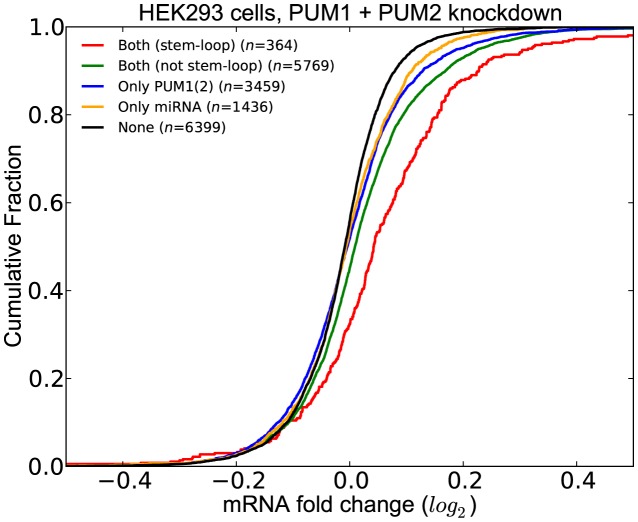
Effect of co-occurrence of PUM and miRNA sites to differential expression of transcripts when PUM1 and PUM2 are knocked down. Transcripts where PUM1(2) and miRNA sites are predicted to be in cooperation with each other (*Both* (*stem-loop*)) have significantly higher LFCs compared to other transcripts.(See Supplementary Table S7 for *P*-values).

Figure 8 shows the comparison of the distribution of LFCs for these five sets of transcripts. We discovered that transcripts in the *Both (stem-loop)* group have significantly higher LFCs as compared to those in the *Both (not stem-loop)* group, the *Only PUM1(2)* group and the *Only miRNA* group. Importantly, the difference between the LFCs of the groups *Only PUM1(2)* and *Both (not stem-loop)* is not significant, indicating that the functional effect of co-occurrence of PUM1(2) and interacting miRNAs is only seen when their sites are located in a stem-loop. The same results are obtained when we repeated the analysis with residuals (Supplementary Figure S7) These results suggests that the cooperation of PUM1(2) and miRNAs is a widespread phenomenon.

#### HuR have potential cooperative interactions with MSI1

**Figure 9.**
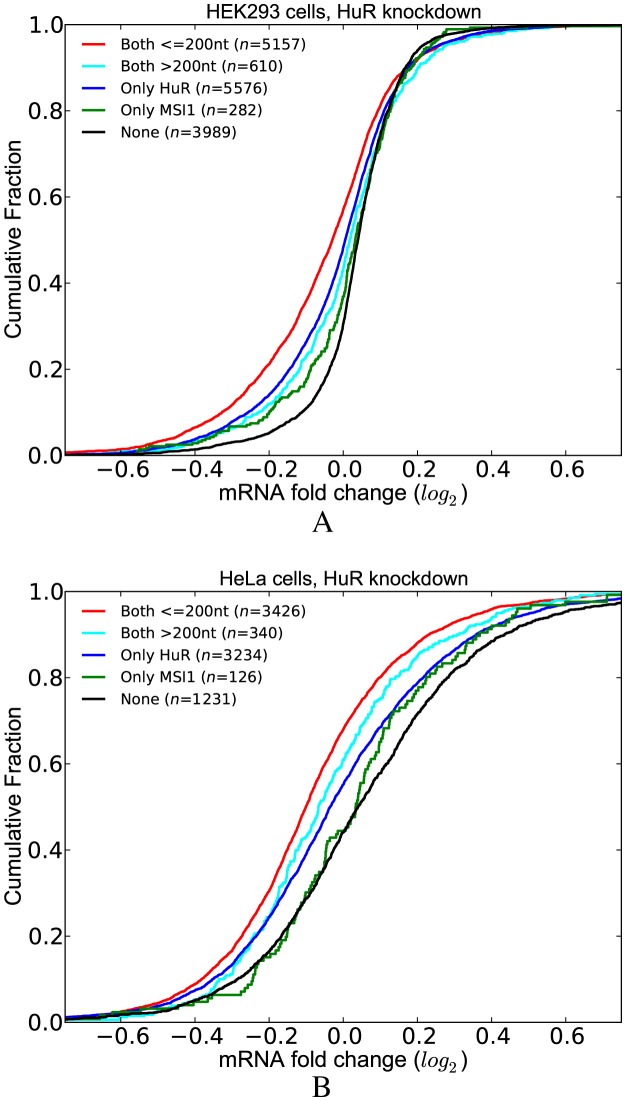
Co-occurrence of nearby HuR and MSI1 sites have a functional effect. Transcripts that have at least one pair of HuR and MSI1 sites within 200 nt have significantly higher LFCs upon HuR depletion. (**A**) HuR knockdown in HEK293 cells. (**B**) HuR knockdown in HeLa cells. (See Supplementary Tables S8 for *P*-values).

Our assessment of the co-occurrence analysis for HuR (Supplementary Figure S4) revealed MSI1 as an RBP whose sites significantly co-occur nearby HuR sites. Since MSI1 is known to increase the stability of its target mRNAs (i.e. similar to HuR), we sought to determine whether this co-occurrence has a functional effect using HuR knockdown datasets. For this purpose, we classified the transcripts into five groups based on whether they contain a HuR or MSI1 site. We then used the HuR knockdown datasets to compare the LFCs of the transcripts in these groups. Figure 9A and B show the distribution of LFCs upon HuR depletion in HEK293 and HeLa cells, respectively. These studies revealed that transcripts that contain a MSI1 site within 200 nt of the HuR site are more destabilized upon HuR depletion. Our results from both datasets indicate that the presence of a MSI1 site proximal to that of HuR increases this effect. These observations were maintained when the analysis was performed with residuals (Supplementary Figure S8).

#### miR-148b have potential cooperative interactions with HNRNPC

**Figure 10.**
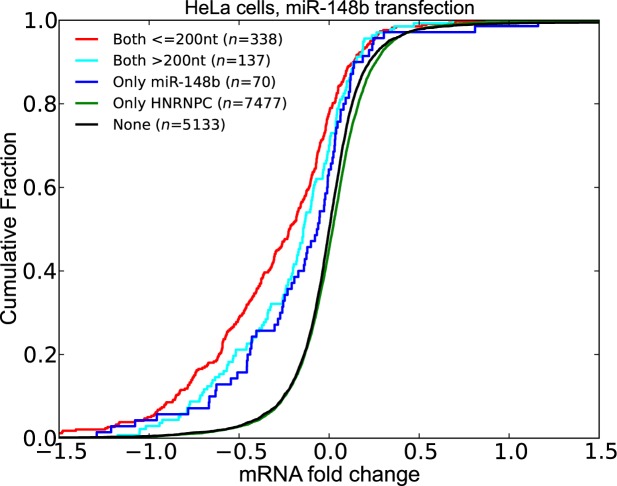
Co-occurrence of miR-148b and HNRNPC sites have a functional effect. Transcripts that have at least one pair of miR-148b and HNRNPC sites within 200 nt have significantly higher LFCs upon miR-148b transfection. (See Supplementary Tables S9 for *P*-values).

We repeated the above analysis for miR-148b using transfection data in HeLa cells (26). HNRNPC is known to decrease the stability of its target mRNAs similar to miRNAs. We classified the transcripts into five groups based on whether they contain a miR-148b or HNRNPC site. We then compared the distribution of LFCs of these sets of transcripts (Figure 10) Similar to our previous observations, we determined that transcripts containing an HNRNPC site within 200 nt of the miR-148b site are more destabilized upon miR-148b transfection. These results indicated that the presence of a nearby HNRNPC site increases the effect of miR-148b.

Similar to our analysis of knockdown datasets, we checked whether 3′UTR length and expression levels are correlated with LFCs in miR-148b transfection dataset. We found a Spearman correlation of 0.04 between LFCs and 3′UTR length and a correlation of −0.02 between LFCs and expression levels. Next, we calculated the residuals of a regression model that predicts LFCs using 3′UTR length and expression levels. Importantly, these findings were also observed when we repeated the analysis with these residuals (Supplementary Figure S9).

### Predicting stability and gene expression with a logistic regression model

**Figure 11.**
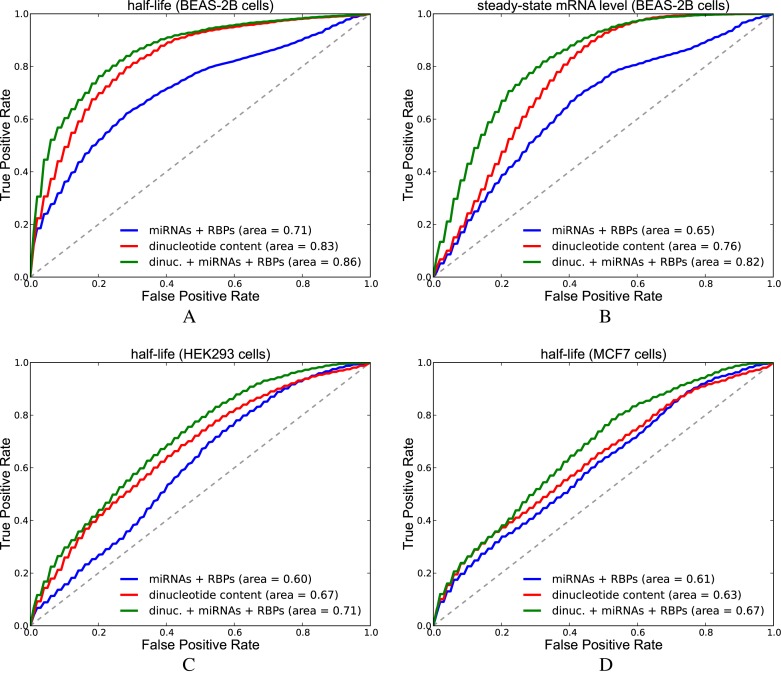
Results of predicting half-life and steady-state mRNA levels in Zhao and Schueler datasets. Figures show ROC curves (interpolation of 100 curves) of logistic regression models. Area shows the average of 100 AU-ROC values. (**A**) We predict half-life in BEAS-2B cells (Zhao dataset). (**B**) We predict steady state mRNA abundance in BEAS-2B cells (Zhao dataset). (**C**) We predict mRNA half-life in HEK293 cells (Schueler dataset). (**D**) We predict mRNA half-life in MCF7 cells (Schueler dataset) (See Supplementary Tables S10 for *P*-values from Wilcoxon sign-rank test).

Next, we took into account the effect of both RBPs and miRNAs to predict transcript stability and expression levels. For this, we used the following datasets: (i) effect of 3′UTR segments to mRNA half-lives and steady-state mRNA abundance in BEAS-2B immortalized human bronchial epithelial cells (Zhao dataset, (28)); and (ii) mRNA half-lives in HEK293 and MCF7 cells (Schueler dataset, (29)). We sorted 3′UTR segments or transcripts based on half-lives or abundance and filtered out those that do not have any miRNA or RBP site. We classified the top 500 transcripts as one class (stable or highly expressed) and the bottom 500 transcripts as the other class (unstable or lowly expressed). We labeled the sequences in the first class with 1 and the second class with 0. Our features consist of dinucleotide counts as well as counts of factor sites where factors have been grouped as *miRNAs, activators* and *repressors*. We defined the *activators* and *repressors* as sets of RBPs that are known to increase or decrease stability in literature, respectively. The *activators* group consisted of the RBPs HNRNPL, PABPC1, PABPC3, PABPC4, PABPC5, PABPN1, RBFOX1, HuR, IGF2BP2 and IGF2BP3, while the *repressors* group consisted of the RBPs CUGBP1, MBNL1, HNRNPC, KHSRP, ZFP36, AUF1, TIA1, PUM1 and PUM2. We used the glmnet package to fit a logistic regression model with L2 regularization. We repeated 10-fold cross validation (CV) 10 times and plotted the interpolated area under ROC curve (AU-ROC) of 100 curves. Figure 11A and B compare the AU-ROC curves of three models that predict half-life or steady-state mRNA abundance in the Zhao dataset, where: (i) *miRNAs, activators* and *repressors* are used as features (miRNAs + RBPs); (ii) counts of 16 2-mers that represent dinucleotide content are used as features (dinucleotide content); (iii) the combination of features used in model (i) and (ii) are used (dinucleotide content + miRNAs + RBPs). We observed that dinucleotide features alone perform much better than features formed from miRNA and RBP binding sites; however, the addition of miRNA and RBP features on top of dinucleotide content features improved the predictive power of the model. Learned parameter values are shown in Supplementary Figure S32. Among the parameters that are learned from the half-life measurements in BEAS-2B cells, the parameter for *activators* has the highest value. The frequency of UU, UC and AA are among the other parameters with highest positive values. On the other hand, the parameter for *repressors* has the lowest value. *miRNAs*, GA, GG, AC content have negative parameter values as well. The parameters learned from steady-state mRNA levels are similar except for the fact that the parameter value for *activators* is much smaller.

Subsequently, we repeated these analyses with half-life datasets in HEK293 and MCF7 cells (Schueler dataset) this time calculating the features considering the 3′UTRs of the transcripts. Figure 11C and D show the ROC curves obtained from the logistic regression models predicting half-life in HEK293 and MCF7 cells, respectively. For both cell types, the increase in AU-ROC between model (i) and model (ii); and between model (ii) and model (iii) was found to be significant according to Wilcoxon signed-rank test (*P*-values are 1.9*E* − 12 and 9.0*E* − 12 for HEK293 cells and 6.8*E* − 05 and 2.7*E* − 13 for MCF7 cells). Our model learned from the half-life dataset from HEK293 cells revealed that the parameter value for *miRNAs* is amongst the lowest values observed together with that of *repressors*, and GA/GG content (Supplementary Figure S32). In the MCF7 model, the effect of *activators* and *repressors* was relatively low.

We also tested whether our features simply represent a bias of 3′UTR length. The 3′UTR segments in Zhao dataset were all of the same length whereas the Schueler dataset contains 3′UTRs of variable length. Predicting half-life with only the 3′UTR length feature generated a mean AU-ROC value of 0.545 and 0.548 in HEK293 and MCF7 cells, respectively. This confirmed that our features do not simply represent 3′UTR length.

In addition to simply counting the number of binding sites as above, we also implemented more advanced features, such as: (i) using the sum of the accessibility of sites of *miRNAs, activators* or *repressors* as features; (ii) considering solely gPARCLIP- or CLIP-supported RBP sites; or (iii) considering the effect of RBP and/or miRNA competition by counting the sites that overlap with other factor sites as 0.5 rather than 1. However, these modifications did not markedly improve the performance of the AU-ROC, with a slight improvement observed in a few cases. This assessment can be found in Supplementary Table S21.

## DISCUSSION

PTR is mediated by the interactions of RBPs and miRNAs with target sites on mRNAs. Recent studies have provided many examples where RBPs and miRNAs function through cooperative or competitive interactions with one another. Despite this fact, the majority of studies have focused on a single factor of interest in isolation from other factors. In this article, we considered the effect of multiple factors concurrently. To achieve this, we mapped all RBP and miRNA binding sites on human 3′UTRs by leveraging existing knowledge generated through *in vitro* (RNAcompete), *in vivo* (CLIP, gPARCLIP) and computational methods (TargetScan, PicTar). We also considered and predicted the secondary structure of 3′UTRs using the computational methods RNAplfold and Sfold.

First, we focused on two well-characterized RBPs, namely HuR and PUM2, and showed that sites that are CLIP-supported are more conserved compared to sites predicted by PFMs and consensus motifs. In line with previous literature (39,41), we found that CLIP-supported sites of HuR are more accessible. In contrast to the expected preference based on the crystal structure of PUM1 (40), we observed that CLIP-supported sites of PUM2 are less accessible. One recent computational study also recovers the same preference for PUM2 (42). Our results on PUM1(2)–miRNA interactions indicate that PUM1(2) is often found in stem-loops. These type of cooperative interactions of PUM1(2) might explain why PUM1(2) sites are located in inaccessible regions according to computationally predicted structures.

To test whether CLIP-supported sites of RBPs displayed any difference as compared to other sites in terms of functional outcome, we compiled HuR knockdown datasets from literature. Since no genome-wide knockdown dataset for PUM1(2) was available, we performed a double knockdown of PUM1 and PUM2 in HEK293 cells to determine differentially expressed transcripts. Our analysis of these datasets revealed that transcripts containing CLIP-supported sites of HuR and PUM1(2) show greater differential expression profiles as compared to other transcripts. To assess the effect of competitive interactions, we compared the LFCs of transcripts where all sites of the RBP of interest are overlapping with sites of other factors to those transcripts that possess at least one site with no overlap. For both HuR and PUM1(2), we found that sites for which there is no competition display greater expected change upon the knockdown of the RBP of interest. To find potential cooperative factors, we also tested whether sites of two factors co-occur more often than expected by random chance. For this, we assessed the interactions of PUM1(2) with target mRNAs, and showed that the transcripts that contain a PUM1(2) site and a site for one of the interacting miRNAs in a stem-loop have higher LFCs compared to transcripts that still contain sites of both of these factors but not in a stem-loop. We also found that there is a distance-dependent cooperative interaction between PUM1(2) and TIA1, HuR and MSI1, and miR-148b and HNRNPC.

We observed that our categorizations of transcripts in the analyses mentioned in the above paragraph results in groups with distinct distributions of 3′UTR length and expression levels. Thus, our observations could be affected by the experimental biases related to these two features. To confirm that we still observe the same results when we remove the effect of 3′UTR length and expression levels, we used these two features in a regression model to predict LFCs in knockdown/transfection datasets. When we repeated our analyses with residuals of these regression models, we observed the same results indicating that our hypotheses still hold when we removed the effect of these potential biases.

Finally, we trained a logistic regression with features compiled from counts of sites of miRNAs, RBPs and dinucleotides to predict mRNA half-life and abundance. The high performance of the dinucleotide-only model is in line with 2013 DREAM5 challenge results where motif models that consider dinucleotide content perform well (43). We hypothesize that dinucleotide features capture the binding motifs of RBPs or miRNAs. Importantly, we also observed that including counts of RBP and miRNAs sites improves the performance in this analysis, and we achieved a 10-fold CV AU-ROC of 0.86 in predicting half-life in BEAS-2B cells. We also considered alternative ways to compile the features: (i) counting RBP sites that are only gPARCLIP or CLIP-supported; (ii) summing the accessibility of sites; and (iii) considering the competition between the factors. However, these modifications did not increase the predictive performance. The first modification may not have improved performance as only 7 out of 19 RBPs that are in *activators* and *repressors* group have CLIP data. Also, counting sites with gPARCLIP support may not result in an accurate estimate of the activity of a specific RBP as the identity of the bound RBP in a gPARCLIP-determined peak is unknown. Lastly, most of the CLIP experiments and gPARCLIP have been performed in HEK293 cells, whereas we predicted transcript half-life and abundance in other cell types, such as BEAS-2B and MCF7 cells. Considering the accessibility of sites will likely improve the performance of these assessments, but is reliant on accurate mRNA secondary structure prediction. While experimental techniques can query mRNA secondary structure *in vivo* (44,45); however, the coverage of the resulting profiles is limited. The recently developed icSHAPE technique (46) has been shown to query the secondary structure of all four bases in mouse ES cells. icSHAPE secondary structure profiles can be utilized in our framework as they become available for human cell types. As such, our assumption that all RBPs and miRNAs prefer binding to accessible regions might be inaccurate. It should be noted that the secondary structure preferences of most of the RBPs are unknown and increased knowledge regarding binding site preferences (aside from nucleotide sequence) would be highly informative.

Here, we have demonstrated the utility of a comprehensive collection of mapped RBP and miRNA binding sites on human 3′UTRs. To the best of our knowledge, this is the first study that accurately predicts transcript half-life and abundance by considering the effects of both RBPs and miRNAs. In addition, the methods presented here provide a novel framework to query cooperative and competitive interactions between trans-acting factors in PTR. Our results on HuR and PUM1(2) knockdown datasets indicate that the presence of multiple, yet proximal RBP and miRNA binding sites must be taken into account to fully understand the PTR of individual transcripts.

## Supplementary Material

SUPPLEMENTARY DATA
