# Supplementary material for: Modeling the combined effect of RNA-binding proteins and microRNAs in post-transcriptional regulation
Source: Nucleic Acids Res. 2016 Feb 2;44(9):e83. doi: 10.1093/nar/gkw048 (PMC4872080; doi:10.1093/nar/gkw048)
Supplement: SUPPLEMENTARY DATA [file supp_44_9_e83__index.html]

Modeling the combined effect of RNA-binding proteins and microRNAs in post-transcriptional regulation — Modeling the combined effect of RNA-binding proteins and microRNAs in post-transcriptional regulation — SUPPLEMENTARY DATA 

# Modeling the combined effect of RNA-binding proteins and microRNAs in post-transcriptional regulation

## SUPPLEMENTARY DATA

- SUPPLEMENTARY DATA
- SUPPLEMENTARY DATA
- SUPPLEMENTARY DATA
- SUPPLEMENTARY DATA
- SUPPLEMENTARY DATA
- SUPPLEMENTARY DATA
