# Supplementary material for: Modeling the combined effect of RNA-binding proteins and microRNAs in post-transcriptional regulation
Source: Nucleic Acids Res. 2016 Feb 2;44(9):e83. doi: 10.1093/nar/gkw048 (PMC4872080; doi:10.1093/nar/gkw048)
Supplement: SUPPLEMENTARY DATA [file supp_gkw048_nar-02849-met-n-2015-File008.pdf]

SUPPLEMENTARY TABLES

Table S1:Primers used for qPCR analysis

FW-PUM1	5'-GACGCTATGGTGGACTACTTCT-3'
REV-PUM1	5'-TGGAACGCACCTGATGTTCTG-3'
FW-PUM2	5'-TCGGGGAATGGGAGAGCTTT-3'
REV-PUM2	5'-GCTGGGACATTGAATGGTGAGA-3'
FW-ACTIN	5'- CATGTACGTTGCTATCCAGGC-3'
REV-ACTIN	5'- CTCCTTAATGTCACGCACGAT-3'
FW-SDAD1	5'-CAATCCATCAAGCCTGCTAGAA-3'
REV-SDAD1	5'-GCGGTTGCATTGCTATCTCTTA-3'
FW-COX10	5'-GCAAGTGTATGATTTGCCAGGA-3'
REV-COX10	5'-TGCAGTGGTACTTACAACCAGA-3'
FW-CDKN1B	5'-TAATTGGGGCTCCGGCTAACT-3'
REV-CDKN1B	5'-TGCAGGTCGCTTCCTTATTCC-3'

Table S2-a: P-values for Figure 2a

**Comparison**	**p-value**
CLIP-supported sites (1-10) vs Other sites	6e-204
CLIP-supported sites (10-50) vs Other sites	0.0
CLIP-supported sites (50-100) vs Other sites	0.0
CLIP-supported sites (1-10) vs CLIP-supported sites (10-50)	2.85e-52
CLIP-supported sites (10-50) vs CLIP-supported sites (50-100)	9e-13
CLIP-supported sites (1-10) vs CLIP-supported sites (50-100)	6e-76

Table S2-b: P-values for Figure 2b

**Comparison**	**p-value**
CLIP-supported sites (1-10) vs Other sites	2.3e-25
CLIP-supported sites (10-50) vs Other sites	5.5e-125
CLIP-supported sites (50-100) vs Other sites	2.1e-201
CLIP-supported sites (1-10) vs CLIP-supported sites (10-50)	0.0
CLIP-supported sites (10-50) vs CLIP-supported sites (50-100)	0.0
CLIP-supported sites (1-10) vs CLIP-supported sites (50-100)	2.25e-06

Table S2-c: P-values for Figure 2c

**Comparison**	**p-value**
CLIP-supported sites (1-10) vs Other sites	3.9e-34
CLIP-supported sites (10-50) vs Other sites	5.5e-127
CLIP-supported sites (50-100) vs Other sites	0.1055
CLIP-supported sites (1-10) vs CLIP-supported sites (10-50)	4e-83
CLIP-supported sites (10-50) vs CLIP-supported sites (50-100)	4.65e-103
CLIP-supported sites (1-10) vs CLIP-supported sites (50-100)	4.1e-33

Table S2-d: P-values for Figure 2d

Comparison	p-value
CLIP-supported sites (1-10) vs Other sites	0.002
CLIP-supported sites (10-50) vs Other sites	7.5e-185
CLIP-supported sites (50-100) vs Other sites	1.85e-287
CLIP-supported sites (1-10) vs CLIP-supported sites (10-50)	2.0e-27
CLIP-supported sites (10-50) vs CLIP-supported sites (50-100)	5.0e-08
CLIP-supported sites (1-10) vs CLIP-supported sites (50-100)	1.0e-22

Table S3-a: P-values for Figure 3a

Comparison	p-value
CLIP-supported sites (1-10) vs Other sites	3.8e-07
CLIP-supported sites (10-50) vs Other sites	2.1e-06
CLIP-supported sites (50-100) vs Other sites	0.006
CLIP-supported sites (1-10) vs CLIP-supported sites (10-50)	0.0475
CLIP-supported sites (10-50) vs CLIP-supported sites (50-100)	0.038
CLIP-supported sites (1-10) vs CLIP-supported sites (50-100)	0.001

Table S3-b: P-values for Figure 3b

Comparison	p-value
CLIP-supported sites (1-10) vs Other sites	2.3e-95
CLIP-supported sites (10-50) vs Other sites	1.55e-126
CLIP-supported sites (50-100) vs Other sites	16e-143
CLIP-supported sites (1-10) vs CLIP-supported sites (10-50)	1.25e-06
CLIP-supported sites (10-50) vs CLIP-supported sites (50-100)	0.3755
CLIP-supported sites (1-10) vs CLIP-supported sites (50-100)	4.45e-06

Table S4-a: P-values for Figure 4a

Comparison with LFCs	p-value
CLIP-supported sites vs Other sites	6e-82
Other sites vs No site	4.1e-13
CLIP-supported sites vs No site	3.75e-97

Table S4-b: P-values for Figure 4b

Comparison	p-value
CLIP-supported sites vs Other sites	7e-15
Other sites vs No site	8.5e-13
CLIP-supported sites vs No site	6e-44

Table S4-c: P-values for Figure 4c

Comparison	p-value
CLIP-supported sites vs Other sites	1.3e-26
Other sites vs No site	6.5e-26
CLIP-supported sites vs No site	9e-55

Table S5-a: P-values for Figure 5a

Comparison	p-value
Competition vs No competition	1.2e-05
Competition vs No site	7.0e-26
No competition vs No site	1.2e-75

Table S5-b: P-values for Figure 5b

Comparison	p-value
Competition vs No competition	7e-13
Competition vs No site	1.94e-08
No competition vs No site	3.71e-49

Table S5-c: P-values for Figure 5c

Comparison	p-value
Competition vs No competition	0.026
Competition vs No site	0.058
No competition vs No site	1.1e-46

Table S6: P-values for Figure 7

Comparison	p-value
Both <= 200nt vs Both > 200nt	0.0005
Both > 200nt vs Only PUM1(2)	0.1255
Both <= 200nt vs Only PUM1(2)	0.0205
Both > 200nt vs Only TIA1	5.5e-08
Both <= 200nt vs Only TIA1	2e-35
Only TIA1 vs None	1.45e-10
Only PUM1(2) vs None	2.6e-05
Both <= 200nt vs None	1.45e-24
Both > 200nt vs None	0.019

Table S7: P-values for Figure 8

Comparison	p-value
Both (stem-loop) vs Both (not stem-loop)	2.45e-09
Both (stem-loop) vs Only PUM1(2)	8.82e-18
Both (not stem-loop) vs Only PUM1(2)	1.61e-15
Both (stem-loop) vs Only miRNA	5.98e-18
Both (not stem-loop) vs Only miRNA	9.61e-11
Only PUM1(2) vs only miRNA	0.37
None vs Both (stem-loop)	9.24e-29
None vs Only miRNA	0.0034
None vs Both (not stem-loop)	2.45e-51
None vs only PUM1(2)	1.98e-05

Table S8-a: P-values for Figure 9a

Comparison	p-value
Both <= 200nt vs Both > 200nt	7.5e-13
Both > 200nt vs Only HuR	0.006
Both <= 200nt vs Only HuR	3.3e-26
Both > 200nt vs Only MSI1	0.113
Both <= 200nt vs Only MSI1	8e-11
Only MSI1 vs None	0.0725
Only HuR vs None	9.5e-50
Both <= 200nt vs None	2.2e-127
Both > 200nt vs None	7e-06

Table S8-b: P-values for Figure 9b

Comparison	p-value
Both <= 200nt vs Both > 200nt	0.001
Both > 200nt vs Only HuR	0.0275
Both <= 200nt vs Only HuR	2.45e-28
Both > 200nt vs Only MSI1	0.0005
Both <= 200nt vs Only MSI1	2.625e-08
Only MSI1 vs None	0.1875
Only HuR vs None	8.5e-15
Both <= 200nt vs None	4.7e-58
Both > 200nt vs None	2.525e-10

Table S9: P-values for Figure 10

Comparison	p-value
Both <= 200nt vs Both > 200nt	0.014
Both > 200nt vs Only miRNA	0.165
Both <= 200nt vs Only miRNA	0.00325
Both > 200nt vs Only HNRNPC	2.05e-15
Both <= 200nt vs Only HNRNPC	2.18e-53
Only HNRNPC vs None	1e-08
Only miRNA vs None	0.000135
Both <= 200nt vs None	2.07e-46
Both > 200nt vs None	8.85e-13

Table S10-a: P-values for Figure 11a

Comparison	p-value
miRNAs + RBPs vs dinucleotide content	6.0e-18
miRNAs + RBPs vs dinuc. + miRNAs + RBPs	5.7e-18
Dinucleotide content vs dinuc. + miRNAs + RBPs	9.8e-18

Table S10-b: P-values for Figure 11b

Comparison	p-value
miRNAs + RBPs vs dinucleotide content	1.3e-17
miRNAs + RBPs vs dinuc. + miRNAs + RBPs	5.7e-18
Dinucleotide content vs dinuc. + miRNAs + RBPs	9.0e-18

Table S10-c: P-values for Figure 11c

Comparison	p-value
miRNAs + RBPs vs dinucleotide content	2.9e-13
miRNAs + RBPs vs dinuc. + miRNAs + RBPs	9.4e-18
Dinucleotide content vs dinuc. + miRNAs + RBPs	3.3e-11

Table S10-d: P-values for Figure 11d

Comparison	p-value
miRNAs + RBPs vs dinucleotide content	1.4e-04
miRNAs + RBPs vs dinuc. + miRNAs + RBPs	7.8e-17
Dinucleotide content vs dinuc. + miRNAs + RBPs	2.7e-13

Table S11-a: P-values for Figure S3a - Residual

Comparison	p-value
CLIP-supported sites vs Other sites	7.01e-38
Other sites vs No site	7.7e-13
CLIP-supported sites vs No site	2.9e-57

Table S11-b: P-values for Figure S3b - Residual

Comparison	p-value
CLIP-supported sites vs Other sites	3.15e-06
Other sites vs No site	0.00014
CLIP-supported sites vs No site	1.25e-14

Table S11-c: P-values for Figure S3c - Residual

Comparison	p-value
CLIP-supported sites vs Other sites	1.1e-20
Other sites vs No site	0.0006
CLIP-supported sites vs No site	1.1e-29

Table S12-a: P-values for Figure S4a - LFCs

Comparison	p-value
CLIP-supported sites vs Other sites	7E-15
Other sites vs No site	8.5E-13
CLIP-supported sites vs No site	6E-44

Table S12-b: P-values for Figure S4b - Residual

Comparison	p-value
CLIP-supported sites vs Other sites	3.13e-06
Other sites vs No site	0.00015
CLIP-supported sites vs No site	1.28e-14

Table S13-a: P-values for Figure S5a - Residual

Comparison	p-value
Competition vs No competition	0.005
Competition vs No site	8e-16
No competition vs No site	3.7e-39

Table S13-b: P-values for Figure S5b - Residual

Comparison	p-value
Competition vs No competition	0.013
Competition vs No site	1.1e-05
No competition vs No site	2.35e-15

Table S13-c: P-values for Figure S5c - Residual

Comparison	p-value
Competition vs No competition	0.060
Competition vs No site	0.059
No competition vs No site	5.8e-27

Table S14: P-values for Figure S6 - Residual

Comparison	p-value
Both <= 200nt vs Both > 200nt	0.0022
Both > 200nt vs Only PUM1(2)	0.00038
Both <= 200nt vs Only PUM1(2)	0.17
Both > 200nt vs Only TIA1	0.0017
Both <= 200nt vs Only TIA1	3.55e-18
Only TIA1 vs None	1.25e-16
Only PUM1(2) vs None	0.0016
Both <= 200nt vs None	0.0042
Both > 200nt vs None	0.014

Table S15: P-values for Figure S7 - Residual

Comparison	p-value
Both (stem-loop) vs Both (not stem-loop)	3.09e-07
Both (stem-loop) vs Only PUM1(2)	3.09e-11
Both (not stem-loop) vs Only PUM1(2)	4.45e-06
Both (stem-loop) vs Only miRNA	2.98e-10
Both (not stem-loop) vs Only miRNA	0.0034
Only PUM1(2) vs only miRNA	0.25
None vs Both (stem-loop)	5.75e-15
None vs Only miRNA	0.1
None vs Both (not stem-loop)	1.05e-10
None vs only PUM1(2)	0.27

Table S16-a: P-values for Figure S8a - Residual

Comparison	p-value
Both <= 200nt vs Both > 200nt	1.32e-11
Both > 200nt vs Only HuR	4.2e-05
Both <= 200nt vs Only HuR	7.98e-12
Both > 200nt vs Only MSI1	0.45
Both <= 200nt vs Only MSI1	1.36e-06
Only MSI1 vs None	0.33
Only HuR vs None	2.47e-26
Both <= 200nt vs None	1.85e-61
Both > 200nt vs None	0.28

Table S16-b: P-values for Figure S8b - Residual

Comparison	p-value
Both <= 200nt vs Both > 200nt	0.03
Both > 200nt vs Only HuR	0.27
Both <= 200nt vs Only HuR	0.003
Both > 200nt vs Only MSI1	0.08
Both <= 200nt vs Only MSI1	0.002
Only MSI1 vs None	0.40
Only HuR vs None	1.66e-08
Both <= 200nt vs None	1.6e-15
Both > 200nt vs None	0.004

Table S17: P-values for Figure S9 - Residual

Comparison	p-value
Both <= 200nt vs Both > 200nt	0.025
Both > 200nt vs Only miRNA	0.20
Both <= 200nt vs Only miRNA	0.01
Both > 200nt vs Only HNRNPC	1.65e-17
Both <= 200nt vs Only HNRNPC	1.2e-54
Only HNRNPC vs None	4.67e-25
Only miRNA vs None	2.95e-05
Both <= 200nt vs None	5.43e-42
Both > 200nt vs None	1.63e-12

SUPPLEMENTARY TABLE LEGENDS

Table S2-17 List of p-values for Figures 2-11 and Supplementary Figures S3-9. P-values in Table S2-S9 and in Table S11-S17 are calculated with one-tailed Mann-Whitney-U test, and p-values in Table S10 are calculated with Wilcoxon signed-rank test.

Table S18. List of RBPs and miRNAs for which the co-occurrence analysis is performed. The factors in this table are selected as they have been previously shown to interact with other factors and/or the consequences of modulating their expression on genome-wide transcript abundance has been tested.

Table S19. Q-values of each factor calculated in co-occurrence analysis. For each factor, there are three sheets that correspond to results from three types of shuffle (i.e., default, AU-content, positional). Rows are all the factors (i.e., all RBPs and miRNAs), and columns correspond to the eight windows (i.e., four windows of length 50 nt on either side). Empirical p-values are calculated by comparing this count to the distribution of counts obtained when factor identities (keeping the site positions fixed) are shuffled 1000 times. q-values are obtained by FDR correction of p-values.

Table S20. Ratios of each factor calculated in co-occurrence analysis. For each factor, there are three sheets that correspond to results from three types of shuffle (i.e., default, AU-content, positional). Rows are all the factors (i.e., all RBPs and miRNAs), and columns correspond to the eight windows (i.e., four windows of length 50 nt on either side). Cell values correspond to the ratios calculated as (the number of co-occurrences in real dataset / the expected number of co-occurrences inferred by averaging the numbers from shuffled datasets).

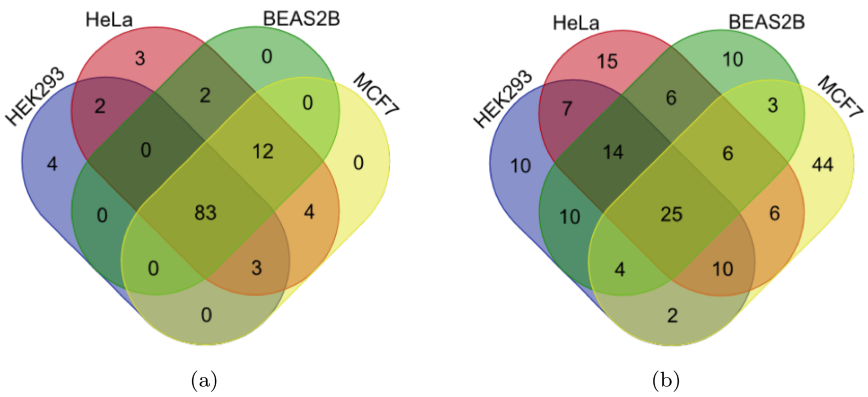

Figure S1: Overlap between the set of expressed RBPs and miRNAs across the cell types a) RBPs b) miRNAs.

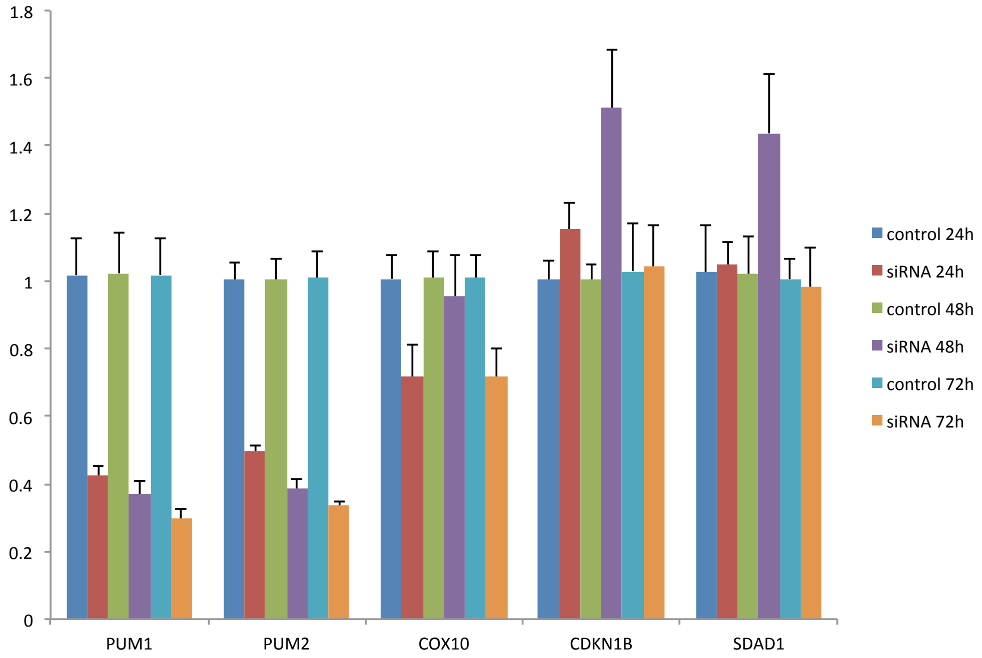

Figure S2: qPCR analysis show that the abundance of PUM1, PUM2 and their known targets decrease after siRNA-mediated PUM1 and PUM2 depletion. Measurements are made at three time points: 24h, 48h, 72h after siRNA transfection. The error bars represent standard error of four replicates.

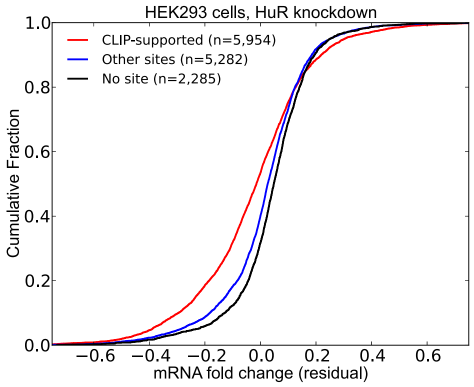

(a)

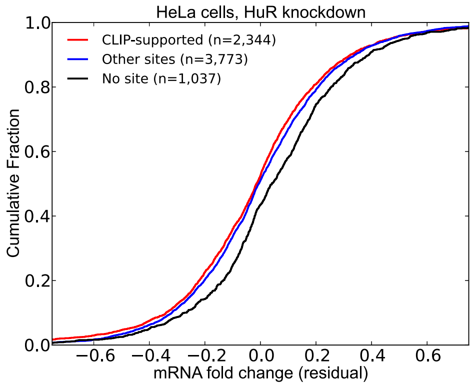

(b)

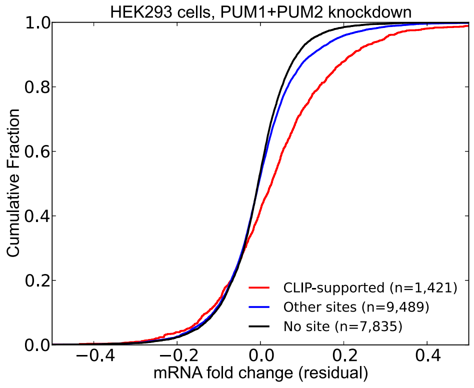

(c)

Figure S3: Comparison of the effect of RBP depletion on transcripts that contain CLIP-supported RBP sites against those that do not contain CLIP-supported RBP sites when the effect of 3'UTR length and expression levels is removed. X-axis shows the residuals of the regression model fit to LFCs using 3'UTR length and expression levels as features. (a) HuR knockdown data in HEK293 cells. (b) HuR knockdown data in HeLa cells. (c) Double knockdown of PUM1 and PUM2 in HEK293 cells. (See Supplementary Table S11 for p-values)

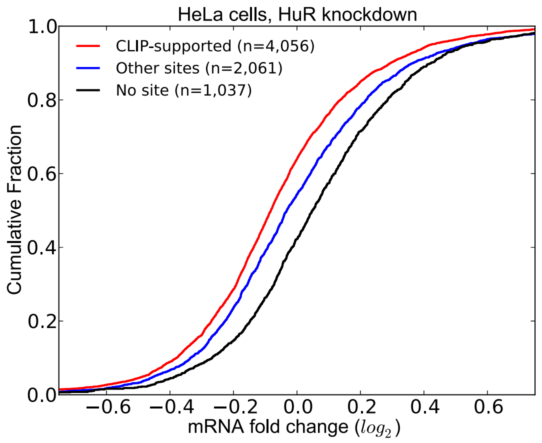

(a)

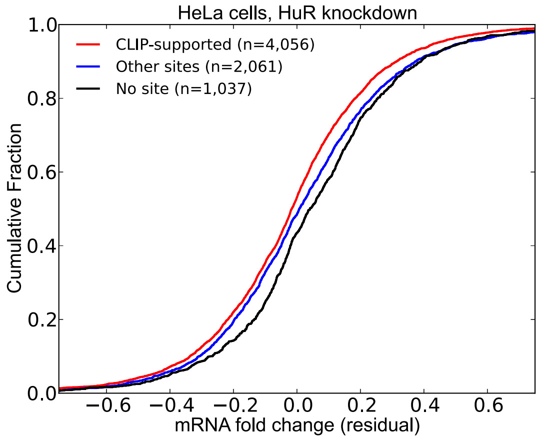

(b)

Figure S4: Comparison of the effect of RBP depletion on transcripts that contain CLIP-supported RBP sites against other transcripts in Lebedeva dataset. CLIP dataset in HEK293 cells is used to define the CLIP-supported sites. (a) X-axis show the LFCs of transcripts. (b) X-axis shows the residuals. (See Supplementary Table S12 for p-values)

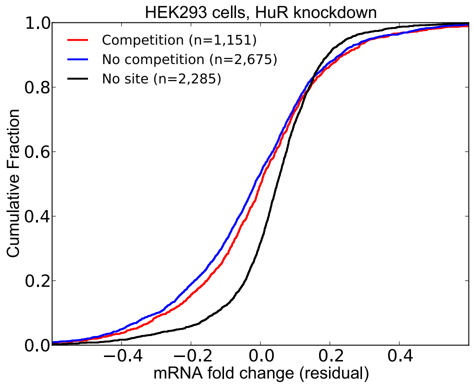

(a)

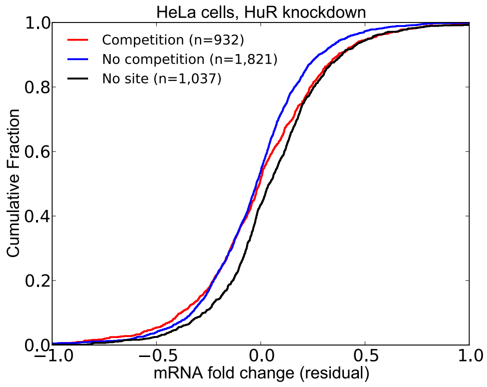

(b)

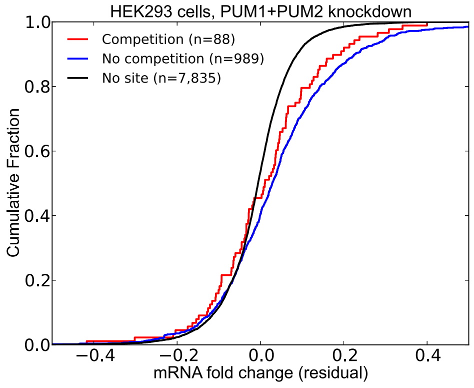

(c)

Figure S5: Functional outcome of the competitive effects of other factors on RBP binding and function when the effect of 3'UTR length and expression levels is removed. X axis shows the residuals from the corresponding regression models. (a) HuR knockdown data in HEK293 cells. (b) HuR knockdown data in HeLa cells. (c) Double knockdown of PUM1 and PUM2 in HEK293 cells. (See Supplementary Table S13 for p-values)

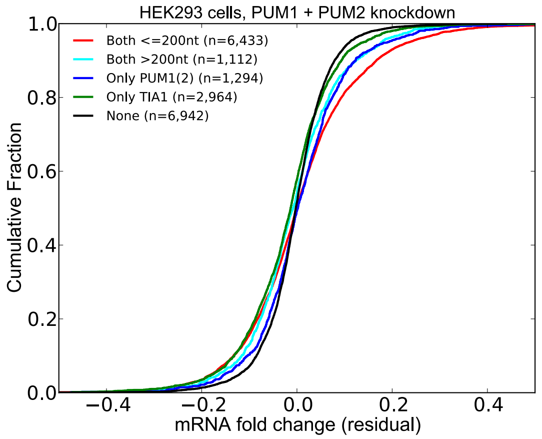

Figure S6: Co-occurrence of PUM1(2) and TIA1 sites still have a functional effect when the effect of 3'UTR length and expression levels is removed. X axis shows the residuals from the regression model. Transcripts that have at least one pair of PUM1(2) and TIA sites within 200 nts have significantly higher residuals upon PUM1(2) depletion. (See Supplementary Table S14 for p-values)

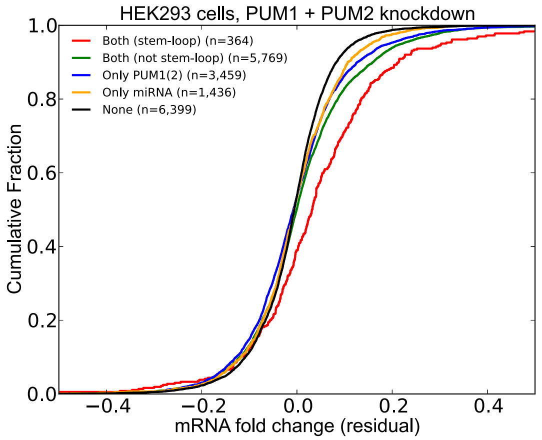

Figure S7: Effect of co-occurrence of PUM and miRNA sites to differential expression of transcripts when PUM1 and PUM2 are knocked down. X axis shows the residuals of the regression model. Transcripts where PUM1(2) and miRNA sites are predicted to be in cooperation with each other (*Both (stem-loop)*) have significantly higher residuals compared to other transcripts.(See Supplementary Table S15 for p-values)

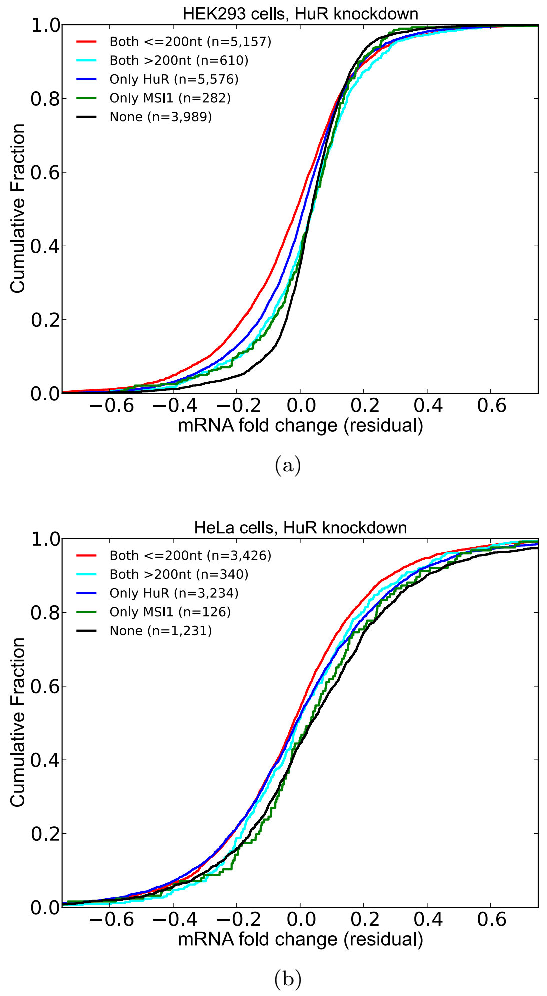

Figure S8: Co-occurrence of nearby HuR and MSI1 sites have a functional effect. X axis shows the residuals of the regression model. Transcripts that have at least one pair of HuR and MSI1 sites within 200 nts have significantly higher residuals upon HuR depletion. a) HuR knockdown in HEK293 cells. b) HuR knockdown in HeLa cells. (See Supplementary Table S16 for p-values)

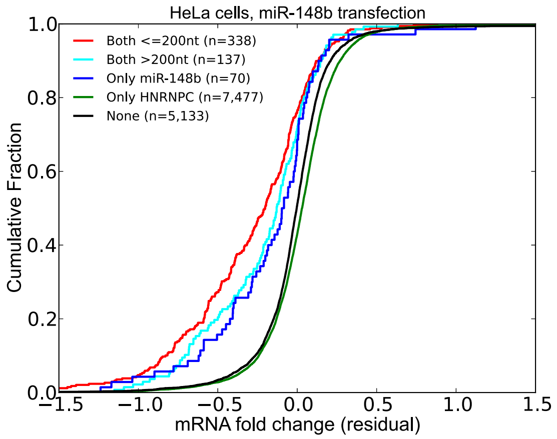

Figure S9: Co-occurrence of miR-148b and HNRNPC sites have a functional effect. X axis shows the residuals of the regression model. Transcripts that have at least one pair of miR-148b and HNRNPC sites within 200 nts have significantly higher residuals upon miR-148b transfection. (See Supplementary Table S17 for p-values)

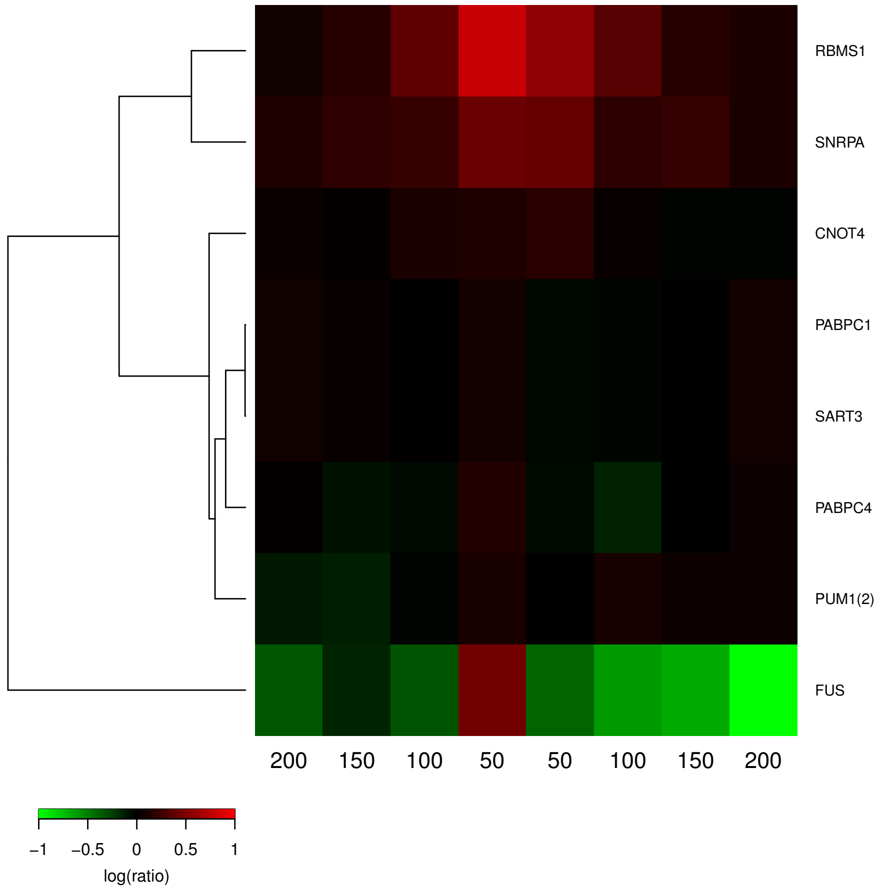

Figure S10: Co-occurrence analysis results for HNRNPL. The set of factors with sites that co-localize with HNRNPL with a q-value < 0.01 in at least one of the windows. Values show the ratio of real co-occurrence counts over expected counts calculated from shuffled data.

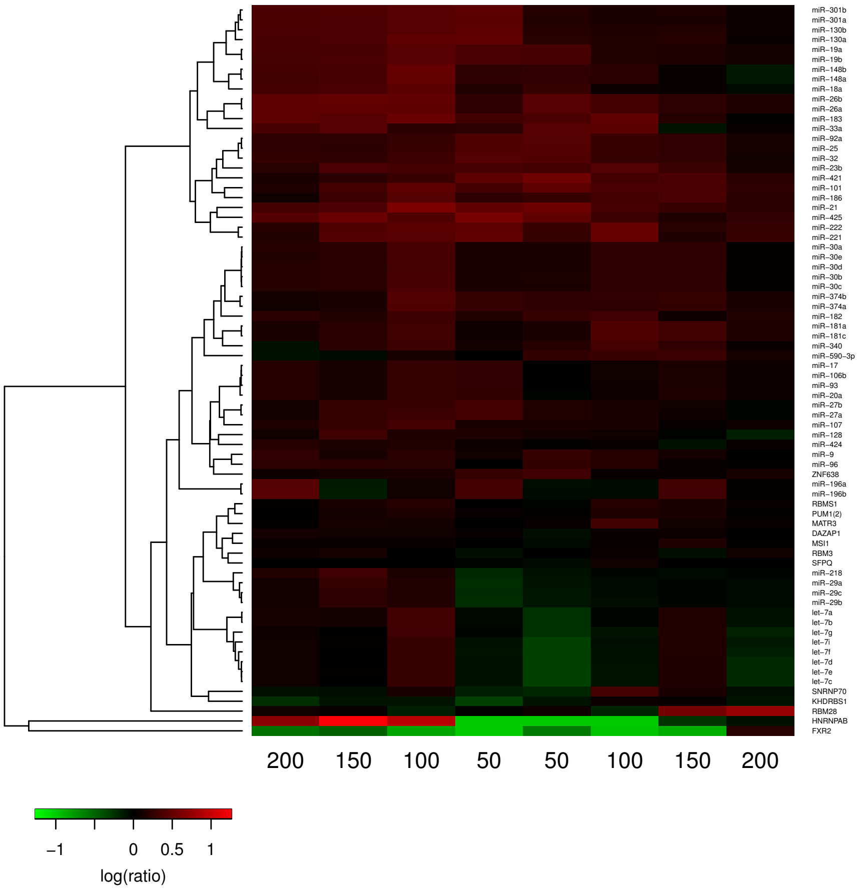

Figure S11: Co-occurrence analysis results for HuR. The set of factors with sites that co-localize with HuR with a q-value < 0.01 in at least one of the windows. Values show the ratio of real co-occurrence counts over expected counts calculated from shuffled data.

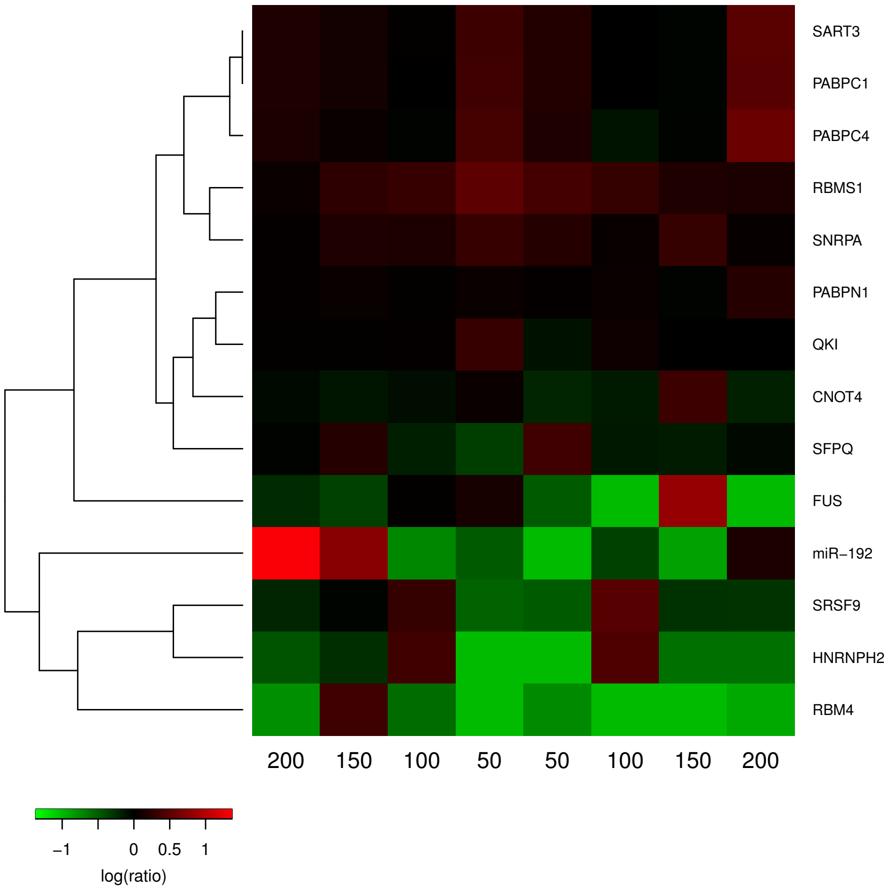

Figure S12: Co-occurrence analysis results for IGF2BP2. The set of factors with sites that co-localize with IGF2BP2 with a q-value < 0.01 in at least one of the windows. Values show the ratio of real co-occurrence counts over expected counts calculated from shuffled data.

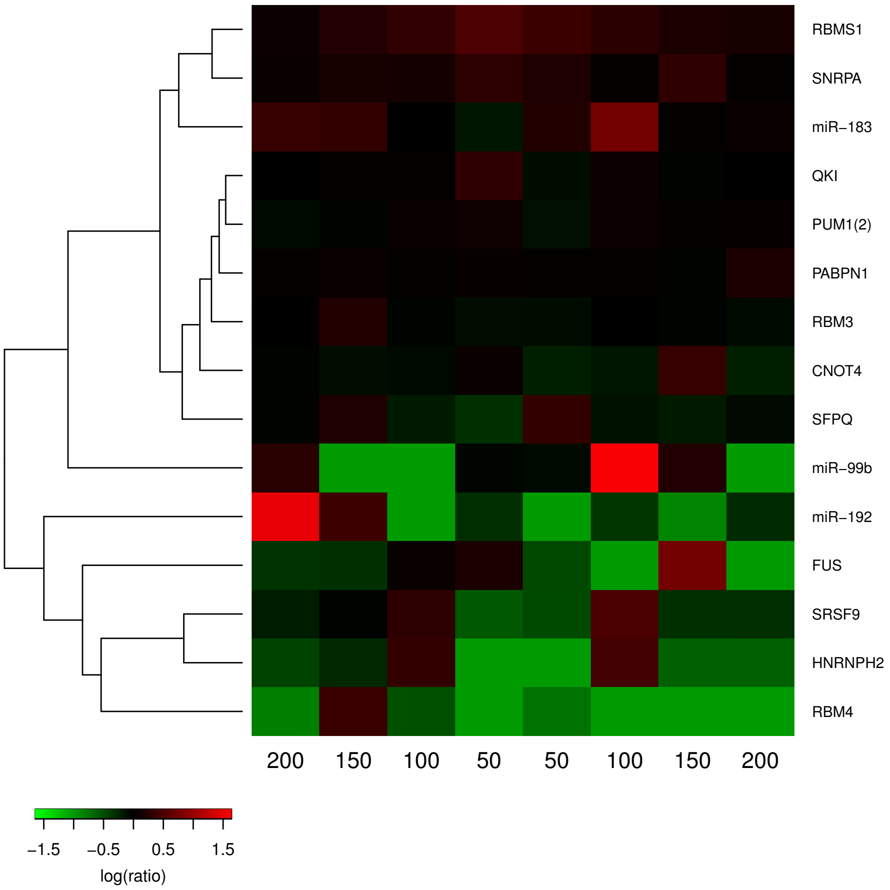

Figure S13: Co-occurrence analysis results for IGF2BP3. The set of factors with sites that co-localize with IGF2BP3 with a q-value < 0.01 in at least one of the windows. Values show the ratio of real co-occurrence counts over expected counts calculated from shuffled data.

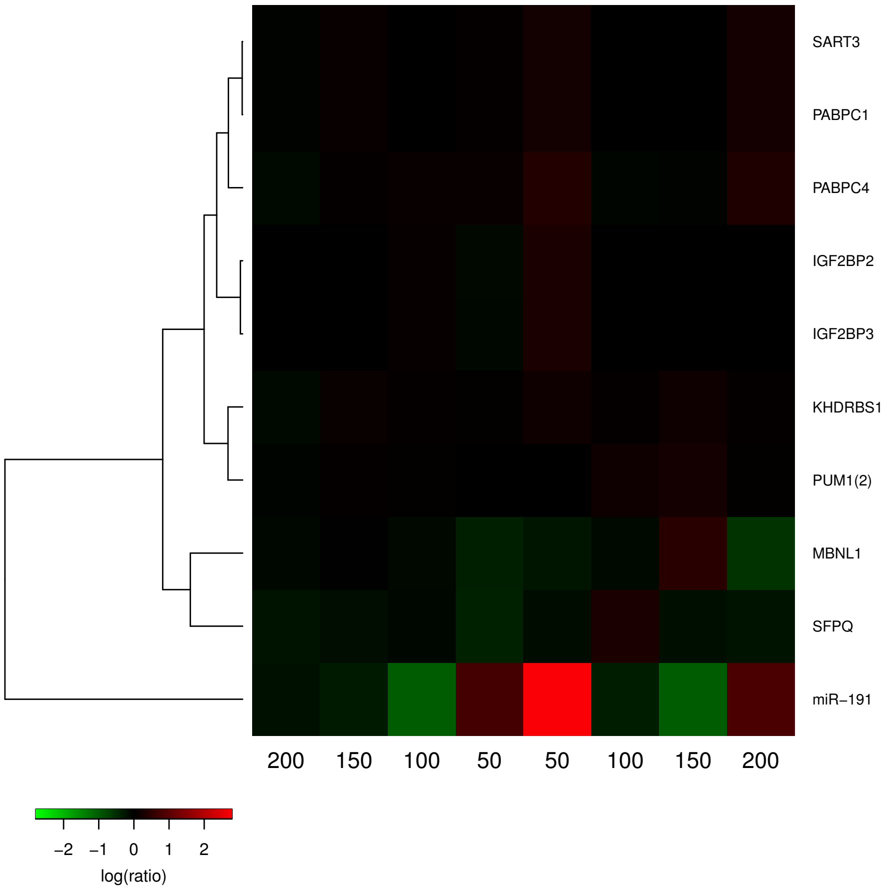

Figure S14: Co-occurrence analysis results for QKI. The set of factors with sites that co-localize with QKI with a q-value < 0.01 in at least one of the windows. Values show the ratio of real co-occurrence counts over expected counts calculated from shuffled data.

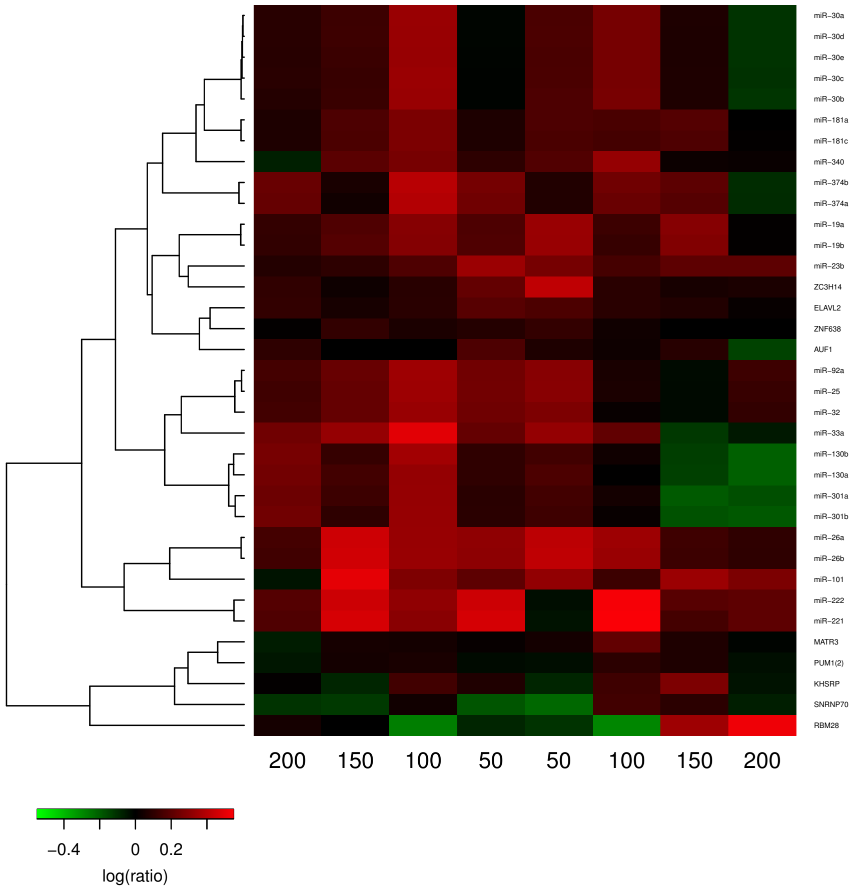

Figure S15: Co-occurrence analysis results for PTBP1. The set of factors with sites that co-localize with PTBP1 with a q-value < 0.01 in at least one of the windows. Values show the ratio of real co-occurrence counts over expected counts calculated from shuffled data.

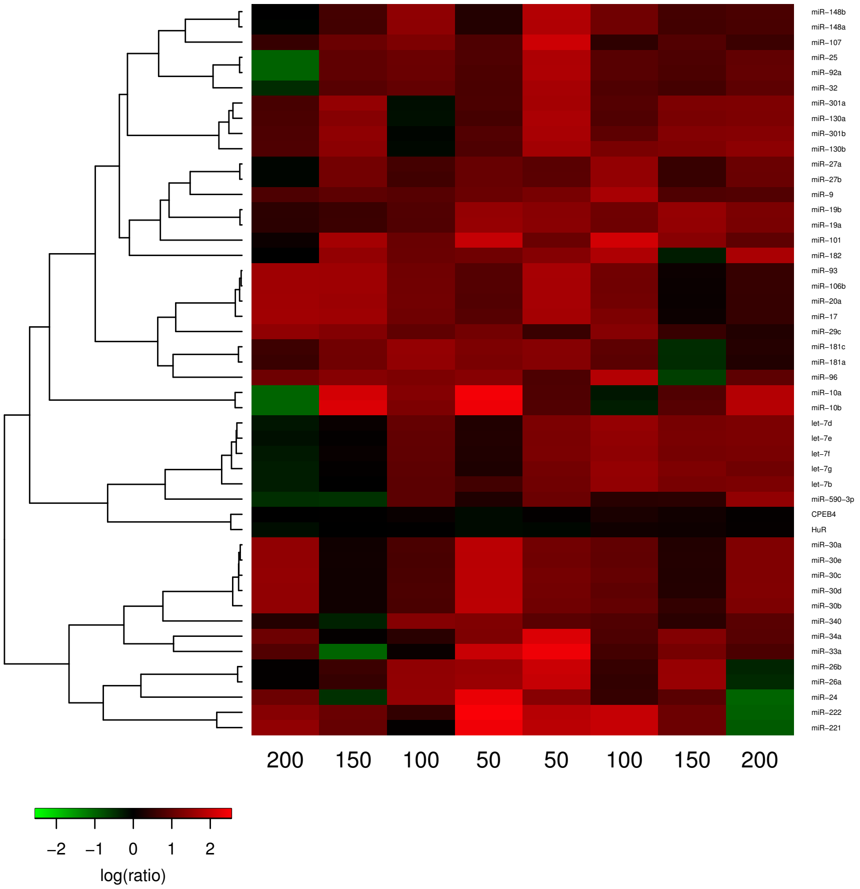

Figure S16: Co-occurrence analysis results for miR-124. The set of factors with sites that co-localize with miR-124 with a q-value < 0.01 in at least one of the windows. Values show the ratio of real co-occurrence counts over expected counts calculated from shuffled data.

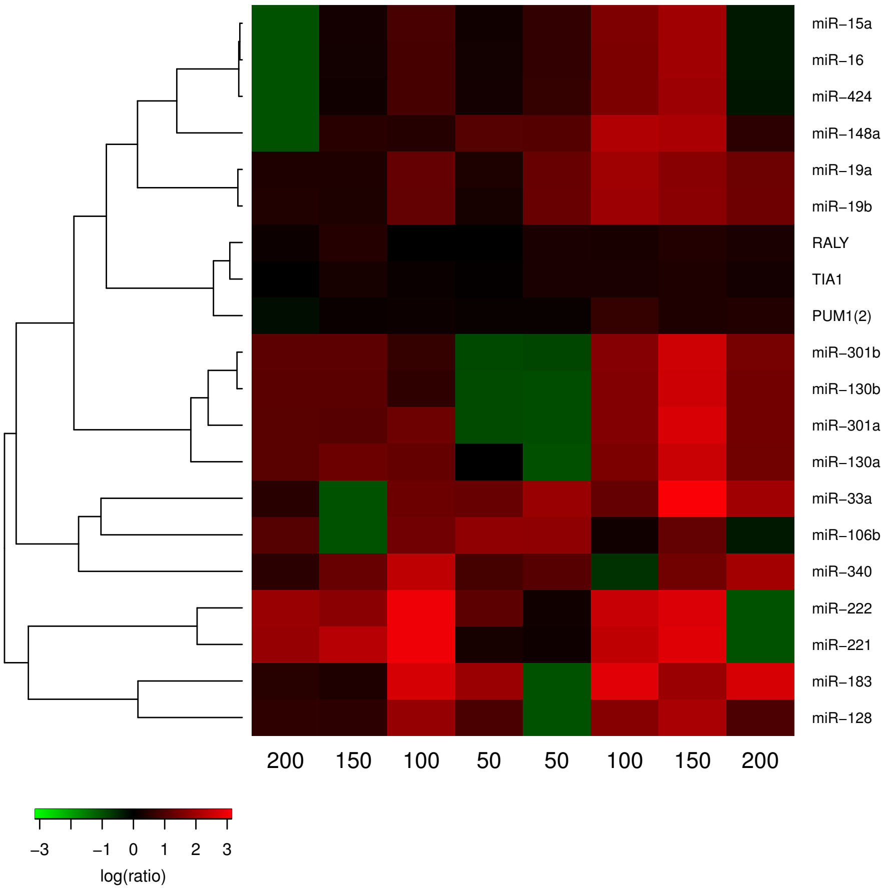

Figure S17: Co-occurrence analysis results for miR-1. The set of factors with sites that co-localize with miR-1 with a q-value < 0.01 in at least one of the windows. Values show the ratio of real co-occurrence counts over expected counts calculated from shuffled data.

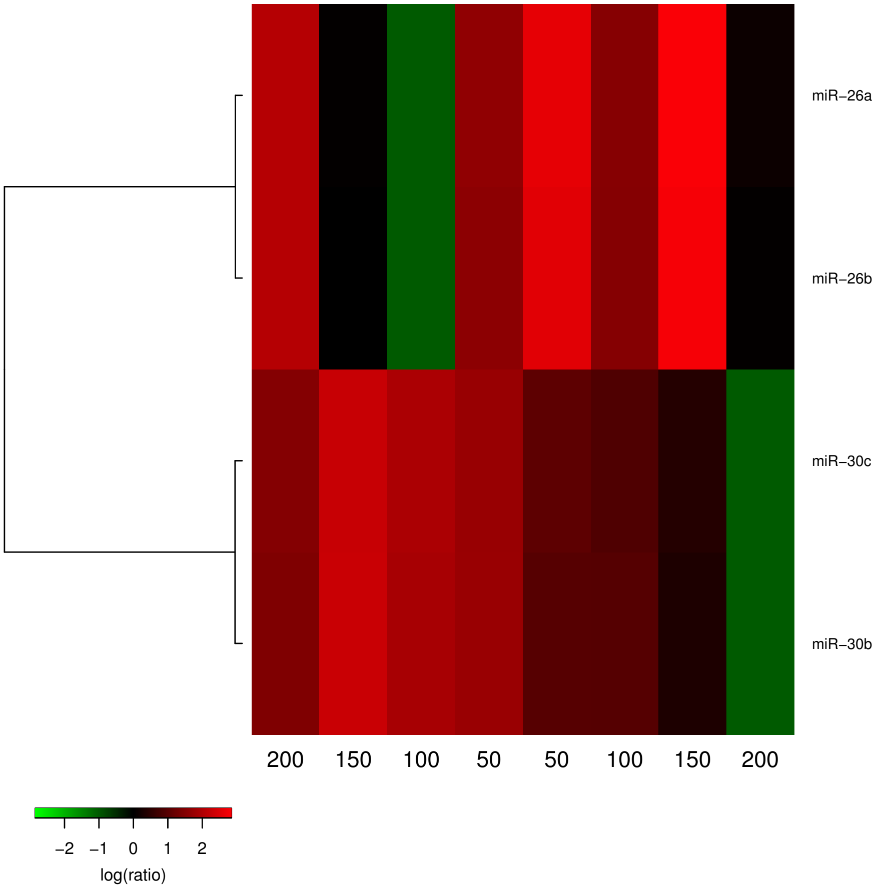

Figure S18: Co-occurrence analysis results for miR-7. The set of factors with sites that co-localize with miR-7 with a q-value < 0.01 in at least one of the windows. Values show the ratio of real co-occurrence counts over expected counts calculated from shuffled data.

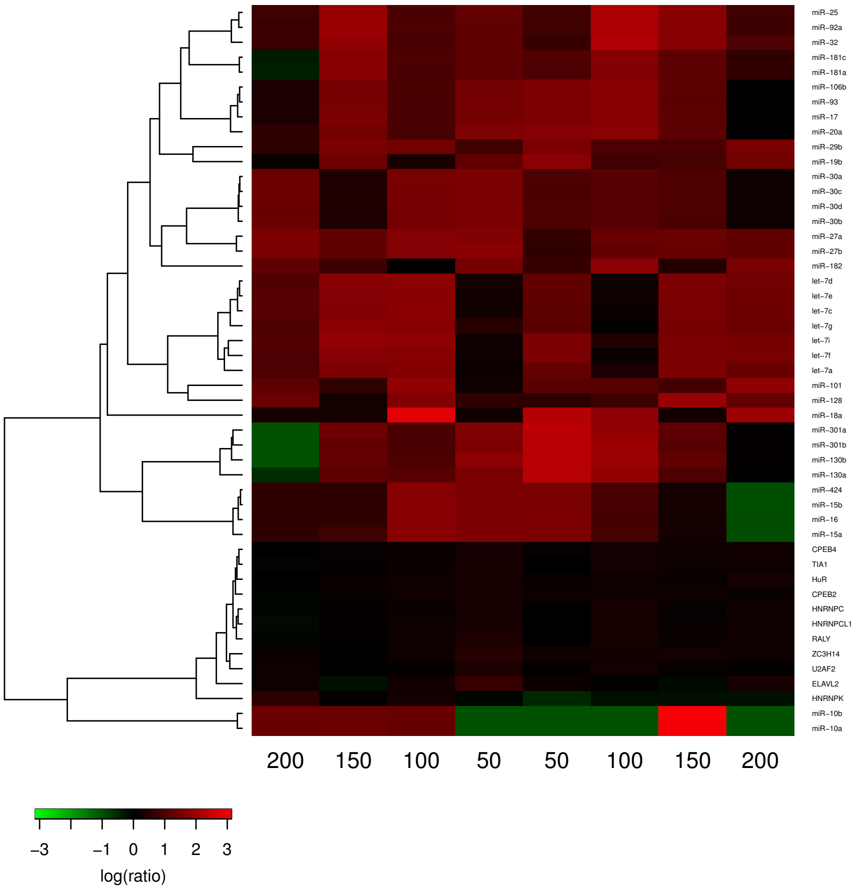

Figure S19: Co-occurrence analysis results for miR-9. The set of factors with sites that co-localize with miR-9 with a q-value < 0.01 in at least one of the windows. Values show the ratio of real co-occurrence counts over expected counts calculated from shuffled data.

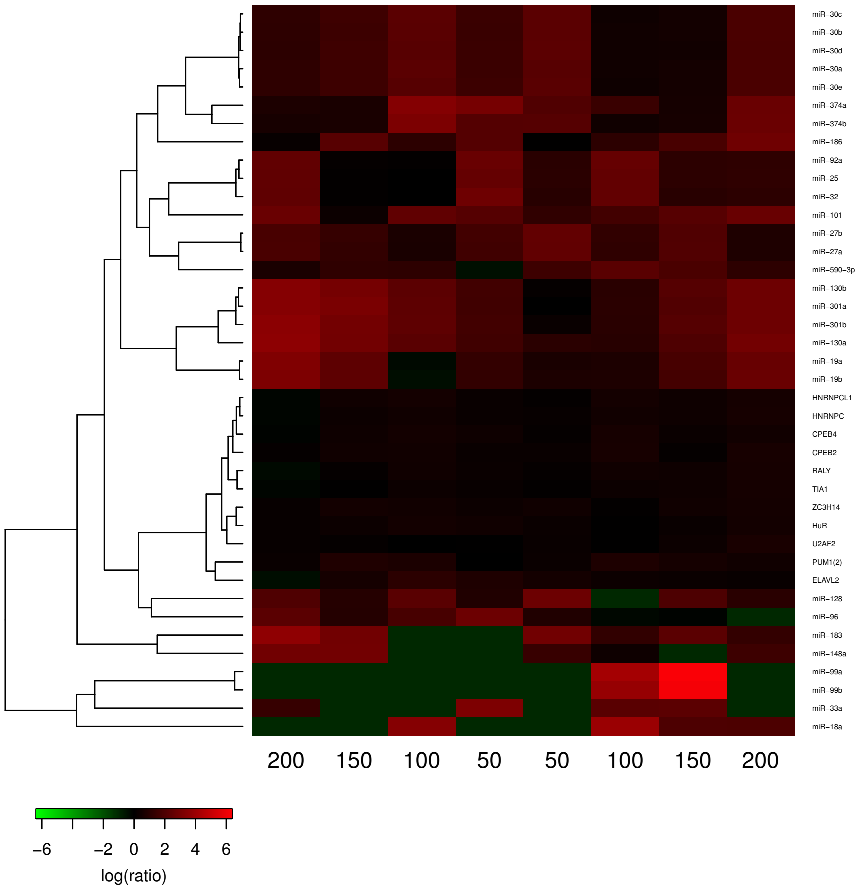

Figure S20: Co-occurrence analysis results for miR-132. The set of factors with sites that co-localize with miR-132 with a q-value < 0.01 in at least one of the windows. Values show the ratio of real co-occurrence counts over expected counts calculated from shuffled data.

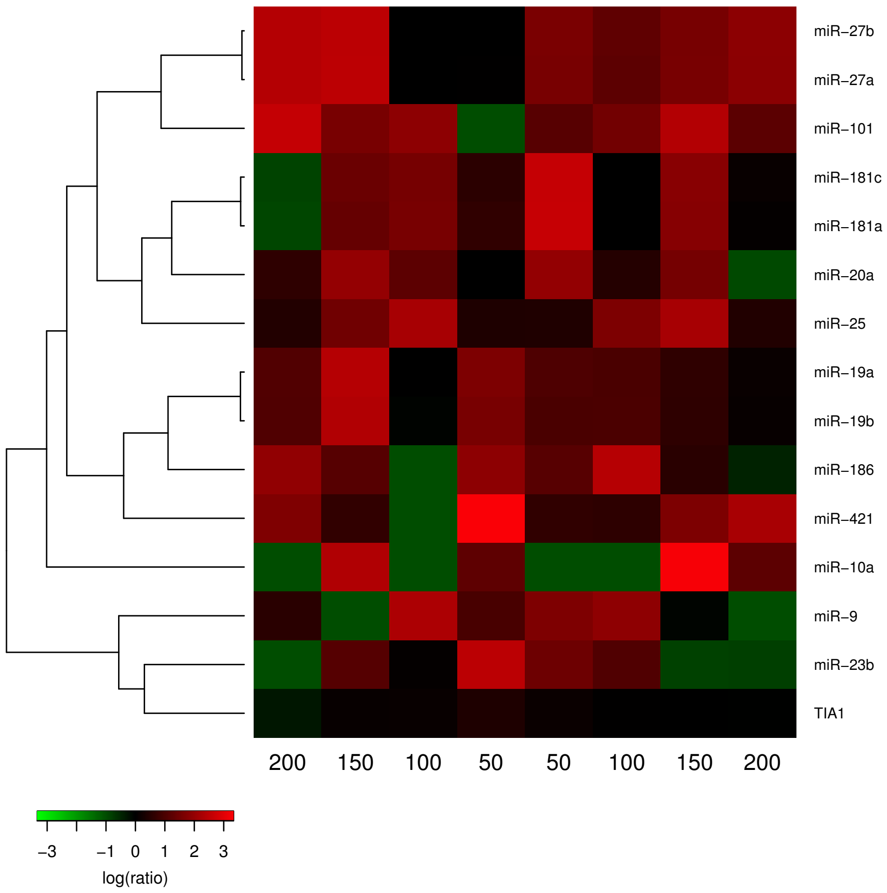

Figure S21: Co-occurrence analysis results for miR-133a. The set of factors with sites that co-localize with miR-133a with a q-value < 0.01 in at least one of the windows. Values show the ratio of real co-occurrence counts over expected counts calculated from shuffled data.

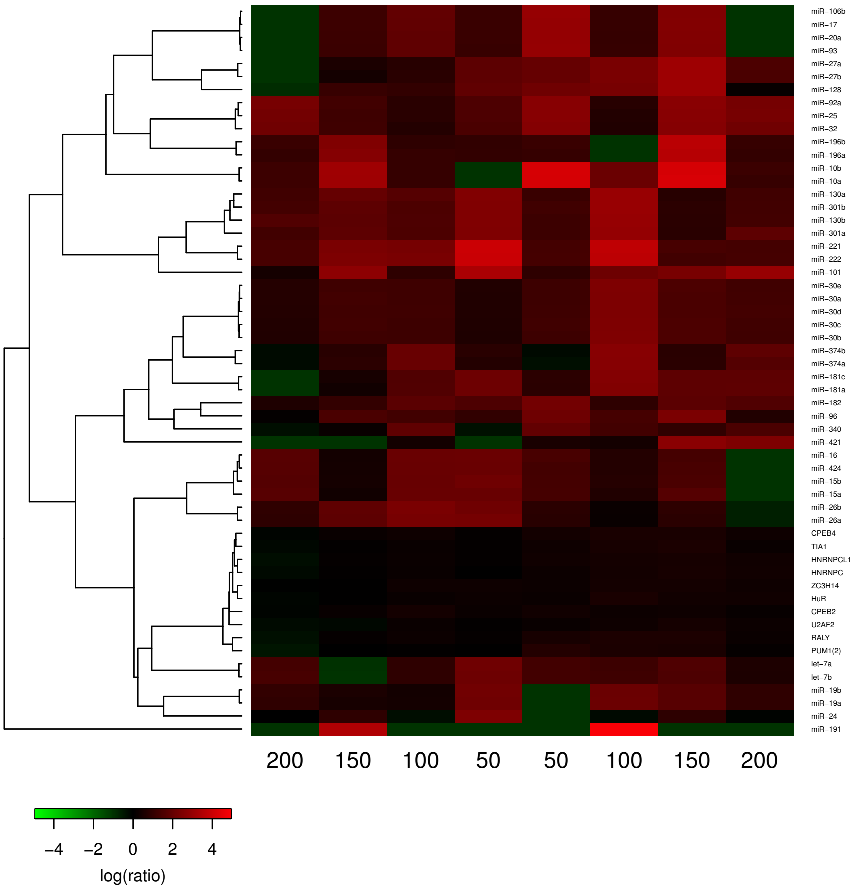

Figure S22: Co-occurrence analysis results for miR-148b. The set of factors with sites that co-localize with miR-148b with a q-value < 0.01 in at least one of the windows. Values show the ratio of real co-occurrence counts over expected counts calculated from shuffled data.

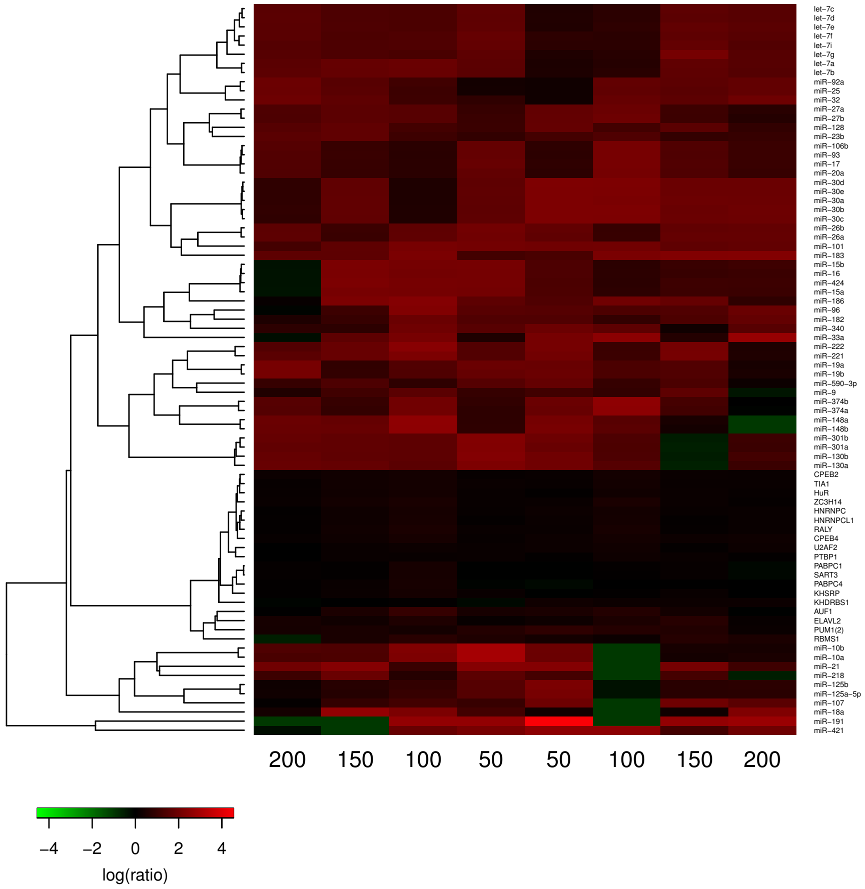

Figure S23: Co-occurrence analysis results for miR-181a. The set of factors with sites that co-localize with miR-181a with a q-value < 0.01 in at least one of the windows. Values show the ratio of real co-occurrence counts over expected counts calculated from shuffled data.

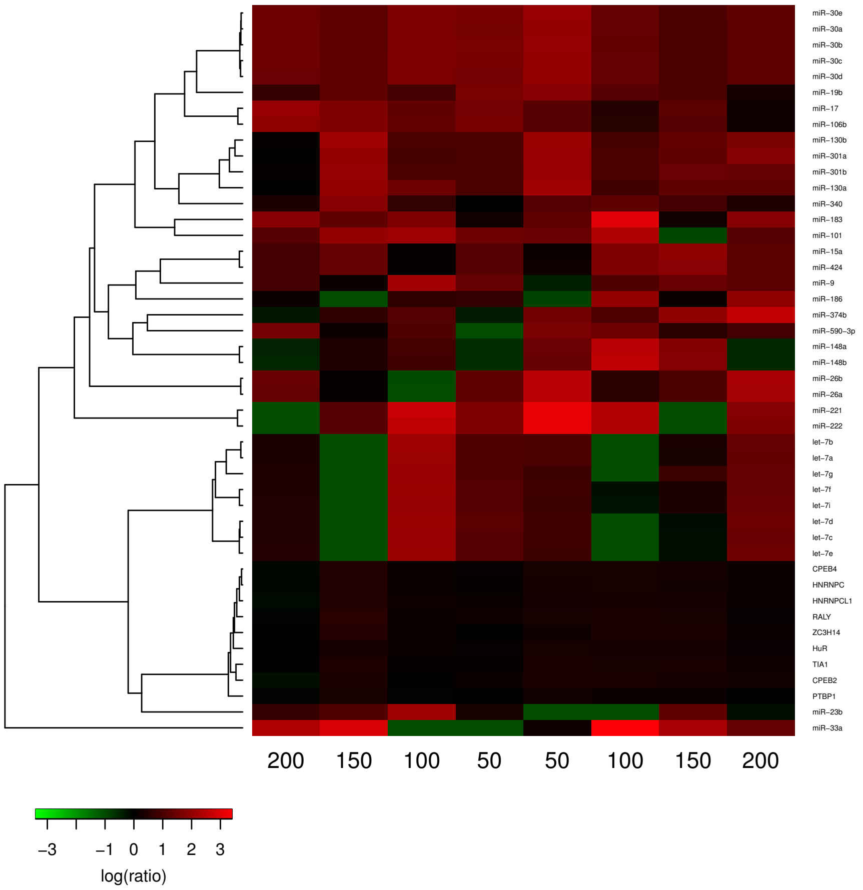

Figure S24: Co-occurrence analysis results for miR-373. The set of factors with sites that co-localize with miR-373 with a q-value < 0.01 in at least one of the windows. Values show the ratio of real co-occurrence counts over expected counts calculated from shuffled data.

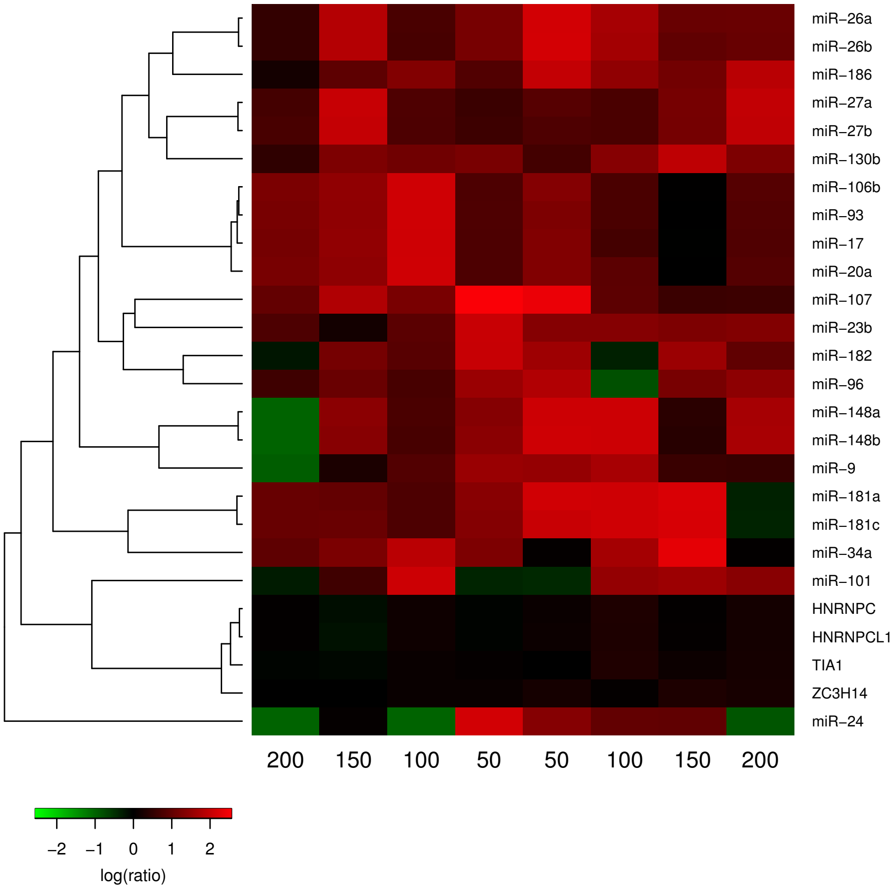

Figure S25: Co-occurrence analysis results for miR-26. The set of factors with sites that co-localize with miR-16 with a q-value < 0.01 in at least one of the windows. Values show the ratio of real co-occurrence counts over expected counts calculated from shuffled data.

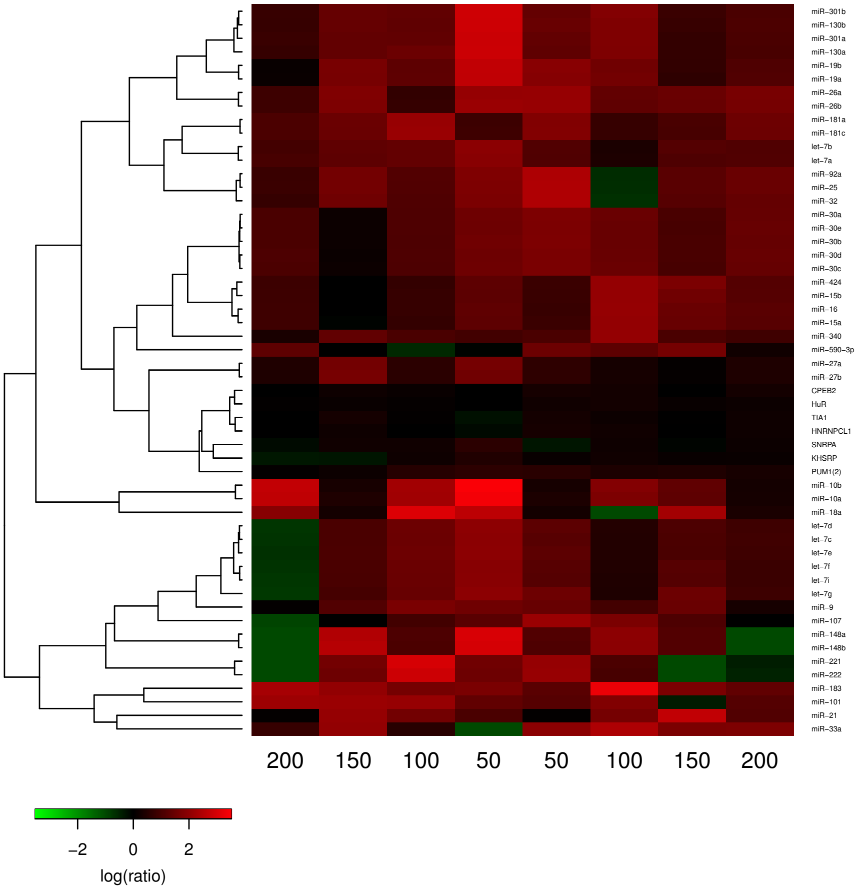

Figure S26: Co-occurrence analysis results for miR-106b. The set of factors with sites that co-localize with miR-106b with a q-value < 0.01 in at least one of the windows. Values show the ratio of real co-occurrence counts over expected counts calculated from shuffled data.

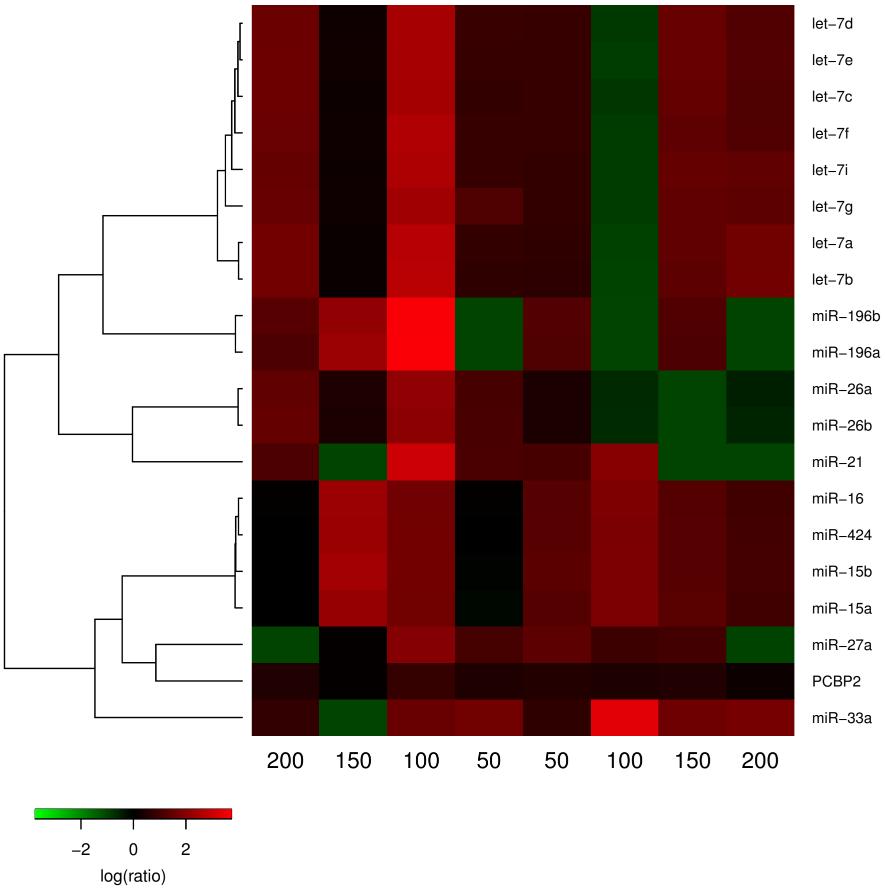

Figure S27: Co-occurrence analysis results for miR-34a. The set of factors with sites that co-localize with miR-34a with a q-value < 0.01 in at least one of the windows. Values show the ratio of real co-occurrence counts over expected counts calculated from shuffled data.

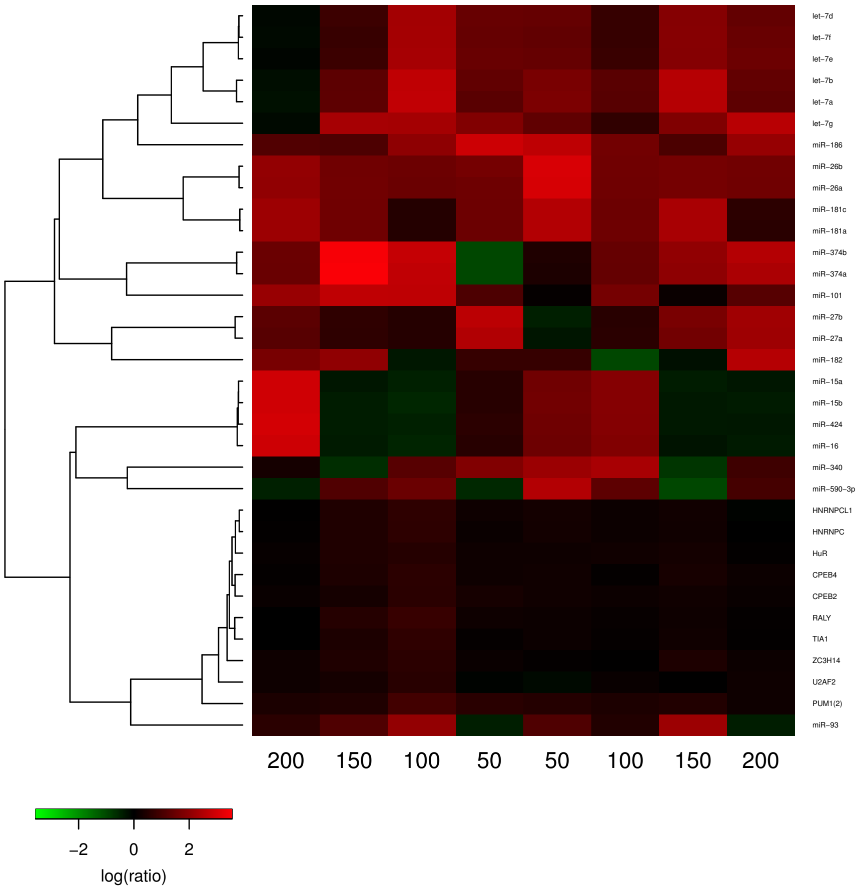

Figure S28: Co-occurrence analysis results for miR-155. The set of factors with sites that co-localize with miR-155 with a q-value < 0.01 in at least one of the windows. Values show the ratio of real co-occurrence counts over expected counts calculated from shuffled data.

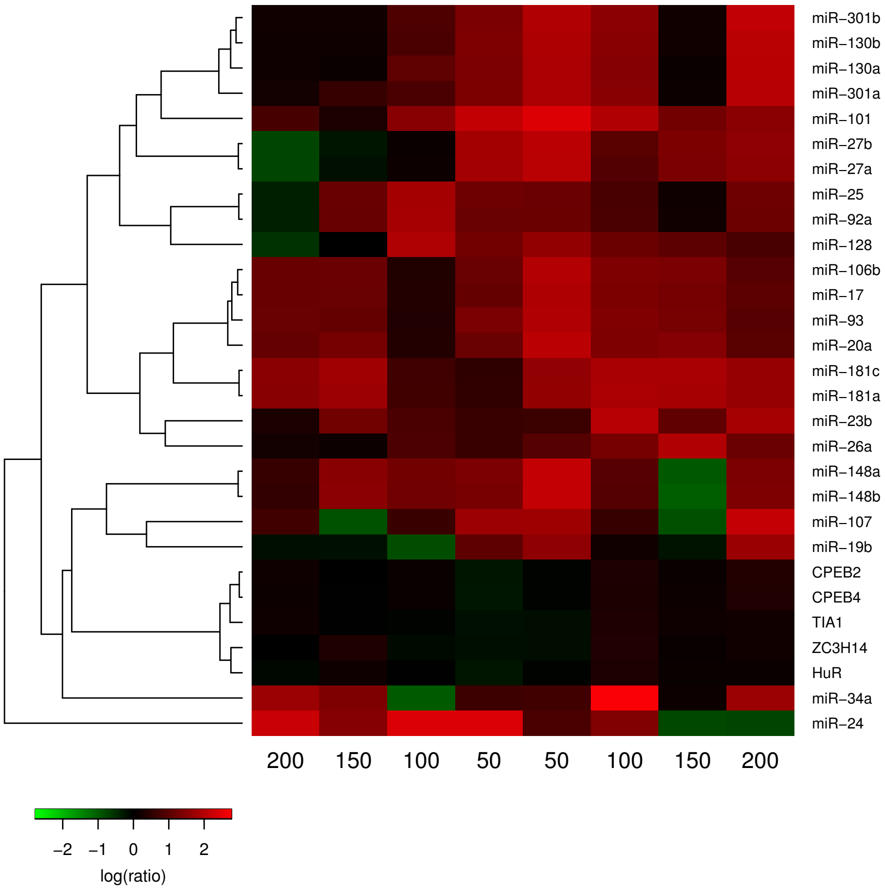

Figure S29: Co-occurrence analysis results for let-7b. The set of factors with sites that co-localize with let-7b with a q-value < 0.01 in at least one of the windows. Values show the ratio of real co-occurrence counts over expected counts calculated from shuffled data.

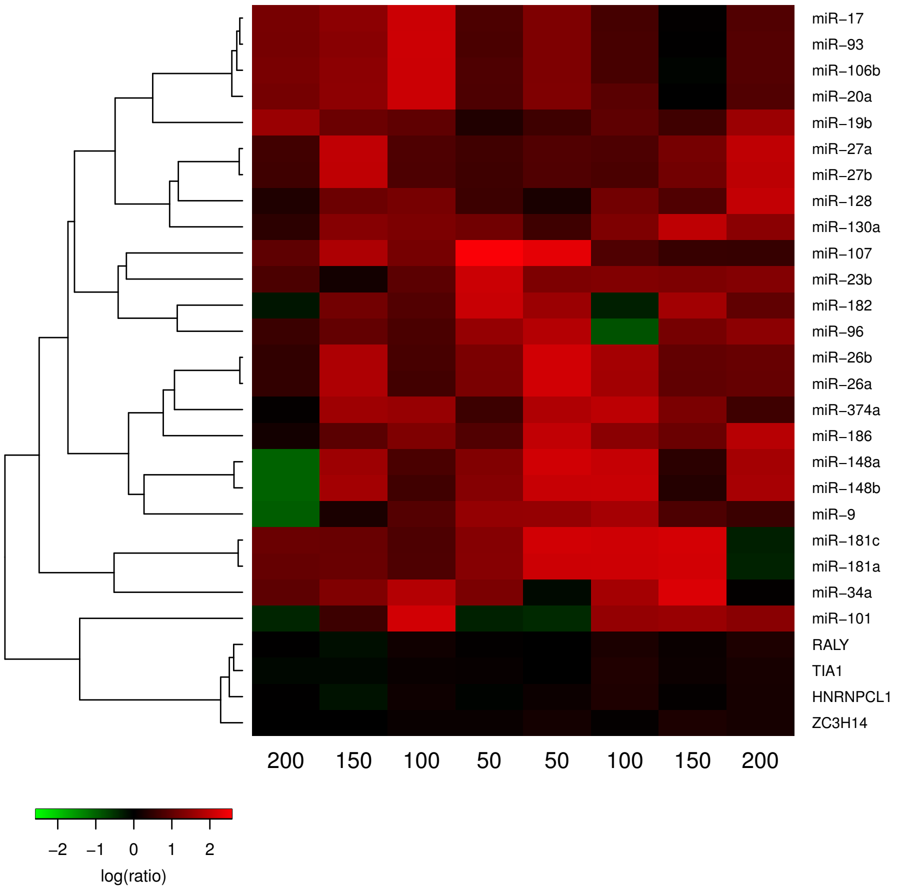

Figure S30: Co-occurrence analysis results for miR-15a. The set of factors with sites that co-localize with miR-15a with a q-value < 0.01 in at least one of the windows. Values show the ratio of real co-occurrence counts over expected counts calculated from shuffled data.

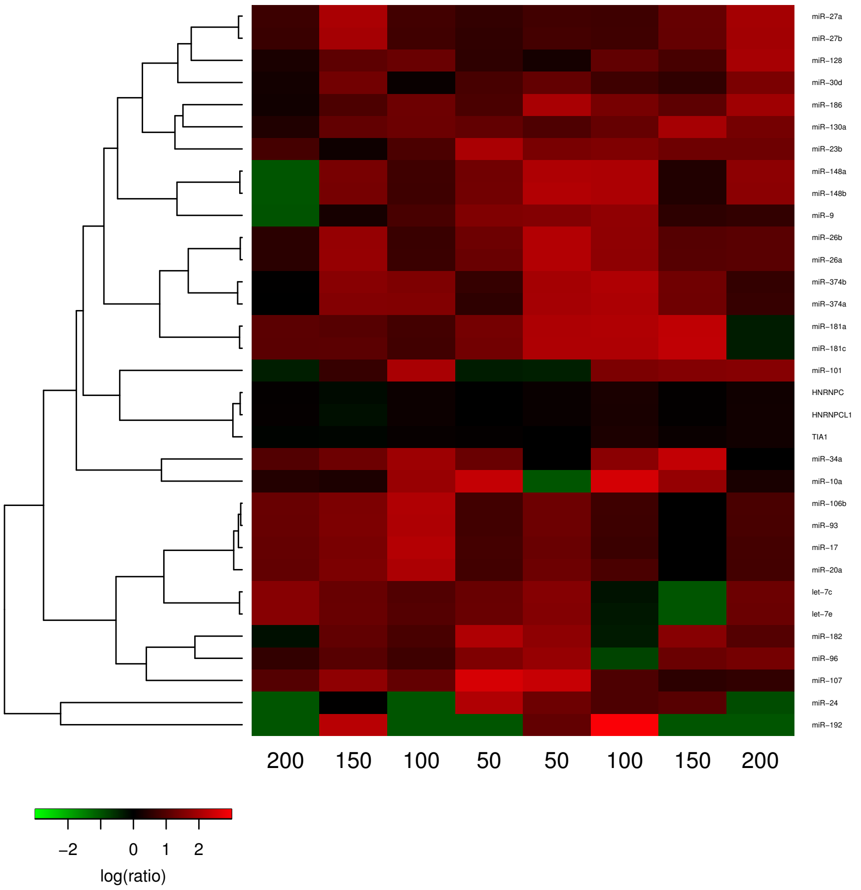

Figure S31: Co-occurrence analysis results for miR-195. The set of factors with sites that co-localize with miR-195 with a q-value < 0.01 in at least one of the windows. Values show the ratio of real co-occurrence counts over expected counts calculated from shuffled data.

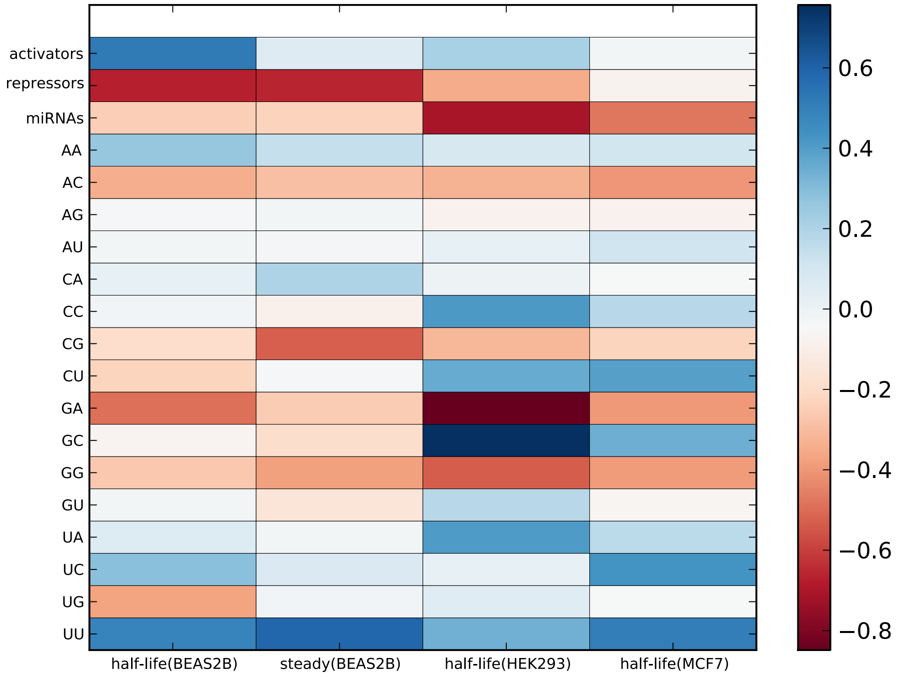

Figure S32: Parameters learned by the logistic regression model from Zhao and Schueler datasets. The rows correspond to the features compiled from RBPs (activators and repressors), miRNAs, and dinucleotides. The columns correspond to half-life and steady-state gene expression datasets from Zhao et al., and half-life datasets from Schueler et al.

experiment	default	gPARCLIP	access (binary)	access(prob)	competition
BEAS-2B (half-life)	0.86	0.85	0.85	0.85	0.86
BEAS-2B (steady)	0.82	0.79	0.80	0.80	0.82
HEK293 (half-life)	0.71	0.71	0.69	0.70	0.70
MCF7(half-life)	0.67	0.67	0.65	0.68	0.67

Table S21: Results of predicting half-life and steady-state mRNA levels in Zhao and Schueler datasets with varying sets of features. Values show the average of the 100 AU-ROC curves. In *gPARCLIP*, only those RBP sites that are located in gPARCLIP- or CLIP-determined peaks are counted. *default* does not take into account the secondary structure information of RBP and miRNA sites whereas *access(binary)* counts only those sites (of RBPs or miRNAs) that have an accessibility value greater than 0.6. *access(prob)* sums the accessibility values of the sites directly. In *competition* mode, sites that overlap with other factors sites are counted as 0.5 rather than 1. These modifications either decreased performance or increased performance very slightly. For predicting half-life and steady-state expression in BEAS-2B cells, *competition* is slightly better than the *default* model (p-values are 1.7e-07 and 0.0014 for prediction of half-life and steady-state expression, respectively). In HEK293 and MCF7 cells, *gPARCLIP* improved over *default* slightly (p-values are 0.015 and 1.3e-07, respectively). In MCF7 cells, taking into account secondary structure with the *access(prob)* model improves the performance (p-value: 3.7e-07). Similarly, *competition* model improves the prediction of half-life in MCF7 cells (p-value: 1.3e-04).
